# Structural basis of the bacterial flagellar motor rotational switching

**DOI:** 10.1038/s41422-024-01017-z

**Published:** 2024-08-23

**Authors:** Jiaxing Tan, Ling Zhang, Xingtong Zhou, Siyu Han, Yan Zhou, Yongqun Zhu

**Affiliations:** 1 Department of Gastroenterology of the Second Affiliated Hospital, School of Medicine and College of Animal Sciences, Life Sciences Institute, Zhejiang University, Hangzhou, Zhejiang China; 2 The MOE Key Laboratory of Biosystems Homeostasis & Protection, and Zhejiang Provincial Key Laboratory of Cancer Molecular Cell Biology, Life Sciences Institute, Zhejiang University, Hangzhou, Zhejiang China; 3 Institute of Microbiology, College of Life Sciences, Zhejiang University, Hangzhou, Zhejiang China; 4 Shanghai Institute for Advanced Study, Zhejiang University, Shanghai, China; 5 Cancer Center, Zhejiang University, Hangzhou, Zhejiang China; 6 Center for Veterinary Sciences, Department of Veterinary Medicine, College of Animal Sciences, Zhejiang University, Hangzhou, Zhejiang China

**Keywords:** Cryoelectron microscopy, Protein aggregation

## Abstract

The bacterial flagellar motor is a huge bidirectional rotary nanomachine that drives rotation of the flagellum for bacterial motility. The cytoplasmic C ring of the flagellar motor functions as the switch complex for the rotational direction switching from counterclockwise to clockwise. However, the structural basis of the rotational switching and how the C ring is assembled have long remained elusive. Here, we present two high-resolution cryo-electron microscopy structures of the C ring-containing flagellar basal body–hook complex from *Salmonella* Typhimurium, which are in the default counterclockwise state and in a constitutively active CheY mutant-induced clockwise state, respectively. In both complexes, the C ring consists of four subrings, but is in two different conformations. The CheY proteins are bound into an open groove between two adjacent protomers on the surface of the middle subring of the C ring and interact with the FliG and FliM subunits. The binding of the CheY protein induces a significant upward shift of the C ring towards the MS ring and inward movements of its protomers towards the motor center, which eventually remodels the structures of the FliG subunits and reverses the orientations and surface electrostatic potential of the α_torque_ helices to trigger the counterclockwise-to-clockwise rotational switching. The conformational changes of the FliG subunits reveal that the stator units on the motor require a relocation process in the inner membrane during the rotational switching. This study provides unprecedented molecular insights into the rotational switching mechanism and a detailed overall structural view of the bacterial flagellar motors.

## Introduction

The bacterial flagellum is a proteinaceous organelle responsible for motility, which is crucial for bacterial infection, biofilm formation and survival in various environments.^1–8^ The flagellum is made up of three distinct parts: the motor, the hook and the filament.^5,9^ The motor is a bidirectional rotary nanomachine that provides the power for the rotation of the flagellum.^3,4,10–13^ The hook functions as a joint that connects the motor to the filament, which acts as a propeller to propel bacteria for swimming in liquid medium and swarming on solid surface.^4^ The motor spans both the inner and outer membranes, and consists of the basal body and several stator units.^14–17^ The basal body is made up of the LP ring, MS ring, C ring, rod and export apparatus. The LP ring acts as a bushing to stabilize the rotation of the rod, which serves as the drive shaft. The MS ring is located on the inner membrane, houses the export apparatus and forms the assembly base for the C ring.^14^ The stator units are ion-conducting channels and generate torque by converting the electrochemical energy from the ion gradient across the bacterial inner membrane into mechanical energy.^14–17^ The flagellar motor can rotate in counterclockwise (CCW) and clockwise (CW) directions, and can switch rapidly between the two rotational states. When all the motors in a bacterial cell rotate in the CCW direction, the filaments form a bundle to propel the cell forward.^4,12,13^ Upon activation of the bacterial chemotaxis pathways, the signal protein CheY is phosphorylated by the kinase CheA and binds to the C ring to switch the rotational direction of the motor from CCW to CW.^3,12,13,18^ Once one or more flagellar motors rotate in the CW state, the filament bundle of the bacterial cell is disassembled, leading to the bacterial cell tumbling and the change of the motility direction.^4,12,13^

The C ring is made up of the proteins FliG, FliM and FliN and attaches to the cytoplasmic face of the MS ring.^3,10,11^ The FliG component contains several subdomains. The C-terminal domain of FliG (FliG_C_), consisting of the FliG_CN_ and FliG_CC_ subdomains, interacts with the stator and mediates torque generation and transmission.^10,11,19^ The middle domain of FliG (FliG_M_) binds to FliM that also interacts with FliN.^10,11^ The N-terminal domain of FliG (FliG_N_) interacts with the C-terminal region of FliF (FliF_C_) and connects the C ring to the MS ring.^20^ However, the C ring is highly fragile and easily dissociates from the flagellar motor during biochemical isolation. Despite that a large number of biochemical studies and structural biology efforts have been made, the detailed structure, assembly and rotational switching mechanisms of the C ring are still obscure.^19–28^ Based on the low-resolution cryo-electron tomography (cryo-ET) analyses of the special flagellar motor in *Borrelia burgdorferi* that harbors the periplasmic flagella, not the general peritrichous or polar flagella, it was proposed that upon binding of the phosphorylated CheY (CheY-P) to FliM and FliN, FliG tilts outward to interact with the outer rim of the stator, resulting in the protomer elongation and outward expansion of the C ring, which induces the rotational direction switching from CCW to CW.^12,25^ However, the outward expansion of the C ring was not observed in other Gram-negative bacteria, and this model is not consistent with the findings in a strain with in-frame deletion of three residues, Pro-Ala-Ala at positions 169–171 of the *Salmonella* FliG, which shortens the sequence, but locks the motor in the CW state.^26^

To uncover the rotational switching mechanism, we purified the particles of the intact C ring-containing flagellar basal body–hook complex from *Salmonella* Typhimurium and determined two cryo-electron microscopy (cryo-EM) structures of the complex, which are in the default CCW and an active CheY mutant-bound CW states, respectively. The structures reveal that the active mutant CheY protein induces significant upward movement of the C ring and inward movement of its protomers, not outward expansion, to mediate the rotational switching, and indicate that the stator units undergo an unexpected relocation process.

## Results

### Overall structures of the C ring-containing motors in the CCW and CW states

**Fig. 1:**
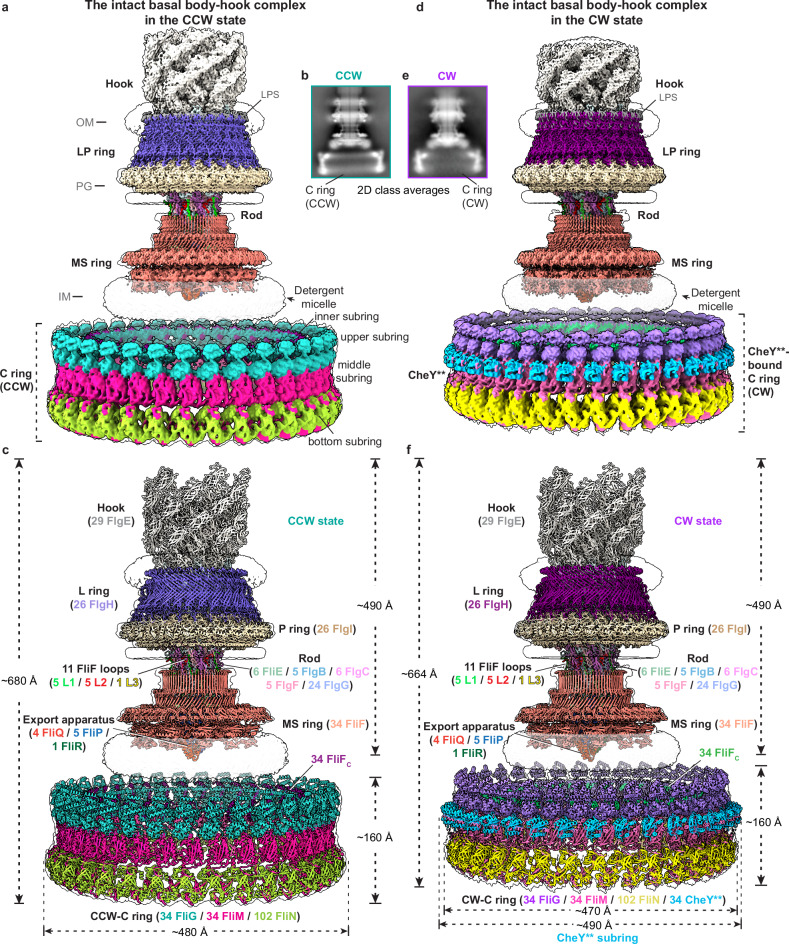
Overall structures of the C ring-containing basal body–hook complex in the CCW and CW states. **a** Composite cryo-EM map of the C ring-containing basal body–hook complex in the default CCW state. The map is constructed by integrating six locally refined density maps (the C ring, the distal rod–hook, the LP ring, the proximal rod–export apparatus, the β-collar–RBM3 subrings and the RBM2–RBM1 subrings of the MS ring) into the globally refined 7.4-Å density map of the basal body–hook complex in the CCW state. OM outer membrane, PG peptidoglycan, IM inner membrane, LPS lipopolysaccharide. **b** Representative 2D class average image of the C ring-containing basal body–hook complex in the CCW state. **c** Overall structure of the C ring-containing basal body–hook complex in the CCW state. The subunits in the complex are colored and labeled as indicated. **d** Composite cryo-EM map of the C ring-containing basal body–hook complex in the CheY**-induced CW state. The map is constructed by integrating six locally refined density maps (the C ring, the distal rod–hook, the LP ring, the proximal rod–export apparatus, and the β-collar–RBM3 subrings and RBM2–RBM1 subrings from the MS ring) into the globally refined 4.3-Å density map of the basal body–hook complex in the CheY**-induced CW state. **e** Representative 2D class average image of the C ring-containing basal body–hook complex in the CW state. **f** Overall structure of the C ring-containing basal body–hook complex in the CheY**-induced CW state.

Given the high fragility of the C ring, we examined several reagents as the media of density gradient centrifugation in the purification of the intact C ring-containing basal body–hook complex in the default CCW state from our previously constructed *ΔfliCD* strain of *S*. Typhimurium,^14^ which lacks the flagellar filament, thus avoiding effects of the filaments on purification of the motor (Supplementary information, Fig. S1a). We found that Percoll, a low-viscosity density gradient medium for use in separating cells, subcellular organelles and viruses, could well protect the structure of the C ring and maintain the integrity of the motor during purification (Supplementary information, Fig. S1). Most purified particles of the basal body–hook complex contain the highly ordered C ring (Fig. 1a, b; Supplementary information, Fig. S1). The cryo-EM density map of the C ring-containing basal body–hook complex in the default CCW state was successfully determined to an overall resolution of 7.4 Å (Fig. 1a; Supplementary information, Figs. S1a, S2a and Table S1). Local refinements of the LP ring, MS ring, rod, hook and export apparatus generated their high-quality cryo-EM density maps at the resolutions ranging from 3.0 Å to 4.3 Å (Supplementary information, Figs. S1a, S2a and Table S1). Initial two-dimensional (2D) classification analyses of manually picked particles revealed that like the MS ring, the C ring adopts a 34-fold symmetric architecture (Supplementary information, Fig. S1a, c). Local refinement of the C ring with C34 symmetry generated a clear density map for the C ring in the CCW state (CCW-C ring) at an overall resolution of 5.9 Å (Fig. 1a; Supplementary information, Figs. S1a, S2a and Table S1). Further local refinement on the four protomers using C34 symmetry-expanded particles improved the density map to a resolution of 4.0 Å (Supplementary information, Figs. S1a, S2). The reconstitution revealed that the structure of the C ring consists of four subrings: the inner, upper, middle and bottom subrings (Fig. 1a, c). The upper, middle and bottom subrings stack in tandem, while the inner subring is relatively separated (Fig. 1a, c). The final structural model of the C ring-containing basal body–hook complex in the CCW state contains 341 subunits from 15 proteins, including 34 FliG, 34 FliM, and 102 FliN subunits in the C ring; 34 FliF subunits in the MS ring; 5 FliP, 1 FliR, and 4 FliQ subunits in the export apparatus; 6 FliE, 5 FlgB, 6 FlgC, 5 FlgF, and 24 FlgG subunits in the rod; 26 FlgH and 26 FlgI subunits in the LP ring; and 29 FlgE subunits in the part of the hook (Fig. 1c). The whole basal body–hook complex has a molecular weight of ~10.2 MDa with a height of ~680 Å, which is much larger than the C ring-free motor (Fig. 1c). The C ring consists of 34 FliG, 34 FliM, and 102 FliN subunits, and has the maximum outer diameter of ~480 Å at the bottom site (Fig. 1c).

To obtain the C ring-containing basal body–hook complex in the CW state, we constructed the constitutively active double mutant D13K/Y106W of CheY (hereafter referred to as CheY**), which can mimic CheY-P to tightly bind to the C ring and lock the rotational direction of the motor in the CW state.^29,30^ The C ring-containing basal body–hook complex in the CW state was obtained by incubating the recombinant CheY** protein with the above purified basal body–hook complex in the CCW state. We successfully determined the cryo-EM map of the basal body–hook complex in the CheY**-locked CW state at an overall resolution of 4.3 Å (Fig. 1d, e; Supplementary information, Figs. S3, S4a and Table S2). The density maps of the LP ring, MS ring, rod, hook, and export apparatus in the CW state were locally reconstructed to the higher resolutions ranging from 2.8 Å to 3.8 Å (Supplementary information, Figs. S3a, S4a and Table S2). 2D classification results showed that the C ring in the CW state (CW-C ring) also possesses a C34 symmetry (Supplementary information, Fig. S3a, c). The density map of the C ring in the CW state was finally refined to an overall resolution of 5.6 Å with C34 symmetry (Fig. 1d; Supplementary information, Figs. S3a, S4a and Table S2). Further local refinement on four protomers with the symmetry-expanded particles improved the density map to a resolution of 4.4 Å (Supplementary information, Figs. S3a, S4). The subunits of CheY** and the CW-C ring have clear densities in the density map (Fig. 1d; Supplementary information, Fig. S4b). There are in all 34 CheY** subunits, which are bound on the outer surface of the middle subring of the C ring and form a CheY** subring structure (Fig. 1d, f). The final structural model of the CheY**-bound basal body–hook complex in the CW state consists of 375 subunits and has a height of ~664 Å with a maximum diameter of ~490 Å at the CheY** subring (Fig. 1f). Although the structures of the LP ring, MS ring, rod, hook, and export apparatus in the CW state are highly similar to those of these components in the CCW state (Fig. 1c, f), the protomers of the C ring in the CW state notably undergo inclination and compaction (Fig. 1c, f), suggesting that the binding of CheY** induces significant conformational changes of the C ring for rotational switching.

In the intact C ring-containing basal body–hook complexes, the MS ring is more stable and harbors much more clear densities for the outer RBM1 subring, outer RBM2 subring and putative inner RBM1 subring,^31,32^ which are made up of 11 RBM1, 11 RBM2 and 23 RBM1 domains of FliF, respectively (Supplementary information, Fig. S5a–c). In the previously determined structure of the C ring-free basal body–hook complex,^14^ 5 L1 and 5 L2 loops, which consist of residues G311–P331 and residues P309–N324 of FliF, respectively, protrude from the β-collar region of the MS ring to bind the surface of the rod for torque transmission. In the intact C ring-containing basal body–hook complexes, there is one more peptide loop L3, which consists of the residues G310–P322 of FliF and is also extended from the β-collar region of the MS ring, bound in a hydrophobic groove that is formed by the α2–α3 linking loop of FliE^6^ with the D0 and D_C_ domains of FlgC^1^ and FlgC^6^ on the surface of the rod (Supplementary information, Fig. S5d–g). The interaction manner of L3 with the rod is different from those of L1 and L2 loops with the rod in the structure of the C ring-free flagellar motor.^14^ Thus, there are totally 11 FliF loops that extend from the MS ring to bind the rod in the basal body for torque transmission. These 11 loops are possibly derived from the FliF subunits in the 11-fold outer RBM1–RBM2 subrings. The more stable MS ring in the C ring-containing basal body–hook complexes indicates that the C ring is also important for the stability of the MS ring during motor assembly.

### Structure of the C ring in the CCW state

**Fig. 2:**
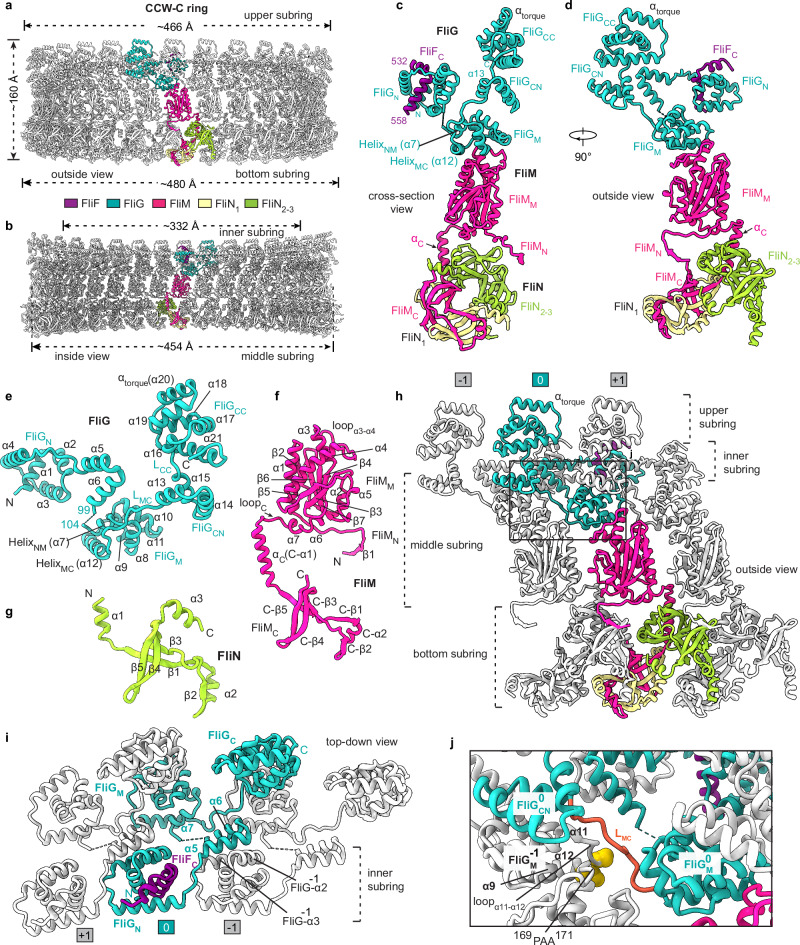
The structure of the C ring in the CCW state. **a**, **b** Side (**a**) and cross-section (**b**) views of the structure of the C ring in the CCW state (CCW-C ring). The subunits of FliG, FliM, FliN_1_, FliN_2–3_ and FliF in a protomer are colored in cyan, red, wheat, green and purple, respectively. **c**, **d** Cross-section (**c**) and side (**d**) views of the protomer of the CCW-C ring. **e**–**g** Structures of the FliG (**e**), FliM (**f**) and FliN (**g**) subunits in the CCW-C ring. **h** Side view of the inter-protomer interactions in the CCW-C ring. Neighboring protomers are colored in gray and numbered sequentially. **i** The inter-subunit interactions of FliG in the CCW-C ring. The FliG_N_ domain extends its α5 and α6 helices to interact with the α2 and α3 helices of FliG^–1^ of the prior protomer. **j** The interactions of the L_MC_ loop with FliG_M_^–1^ in the CCW-C ring. The residues P169, A170 and A171 of FliG_M_^–1^ are shown as yellow spheres.

The C ring in the CCW state has a height of ~160 Å with the minimum inner diameters of ~332 Å at the inner subring, and consists of 34 protomers (Fig. 2a, b; Supplementary information, Fig, S6a). Each protomer is assembled by FliG, FliM and FliN with a stoichiometry of 1:1:3 (Fig. 2c, d). FliG in the CCW-C ring consists of the domains FliG_N_, FliG_M_, FliG_CN_ and FliG_CC_ and adopts a twisted “V”-shaped architecture (Fig. 2e), which is distinct from the extended apo structure of FliG from *A. aeolicus* (Supplementary information, Fig. S6b).^19^ FliM consists of three domains, FliM_N_, FliM_M_ and FliM_C_, and adopts a lifting tong-like structure with FliM_N_ and FliM_C_ as arms (Fig. 2f). FliN is composed of three α-helices and five β-strands, and has a structure similar to FliM_C_ as described (Fig. 2g).^23^ In the C ring, the upper subring is composed of FliG_CC_, while the middle subring is assembled by FliG_CN_, FliG_M_ and FliM_M_ (Fig. 2a–d, h). The bottom subring is formed by FliM_N_, FliM_C_ and FliN (Fig. 2a–d, h). The relatively separate inner subring is constituted by FliG_N_ and FliF_C_ (Fig. 2i).

The protomer of the C ring has a “Y”-shaped architecture (Fig. 2c, d), and obliquely binds to the neighboring two protomers in a “S”-like manner (Fig. 2h), which generates extensive inter-protomer interactions within the C ring. In one protomer, the helices α5 and α6 of FliG_N_ pack onto helices α2 and α3 of the FliG^–1^ subunit of the prior protomer (Fig. 2i). A flexible loop between α6 and Helix_NM_ (α7), which is disordered in the structure, links FliG_N_ to the helix α7 of FliG_M_ (Fig. 2e, i). FliG_M_ binds to the upper surface of FliM_M_ via the α9, α11 and Helix_MC_ (α12) helices and the loop_α8–α9_ (Fig. 2c, e; Supplementary information, Fig. S6c). The following FliG_CN_ domain extends outwards and binds onto the upper surface of the FliG_M_ domain in the prior protomer (Fig. 2h, j). The long linking loop L_MC_, which connects FliG_M_ and FliG_CN_, binds into a cleft between α9 and loop_α11–α12_ of the FliG_M_^–1^ domain, close to the residues P169-A170-A171 (^169^PAA^171^) (Fig. 2j), the deletion of which caused the CW-biased rotation of the motor,^33^ suggesting that the interactions play a key role in the rotational switching. The α15 helix of FliG_CN_ and the following hinge loop L_CC_, which is formed by the conserved M_233_FLF_236_ motif,^10^ brace the FliG_CC_ domain via hydrophobic interactions and stabilize its orientation in the upper region of the protomer (Supplementary information, Fig. S6d). The α_torque_ (α20) helix of FliG_CC_, which interacts with the MotA subunits of a stator unit in torque transmission,^10^ lies on the top of the C ring (Fig. 2h; Supplementary information, Fig. S6e). The stator-interacting charged residues, R281 and D288/D289, are located at the N- and C-terminus of α_torque_,^34^ respectively (Supplementary information, Fig. S6e). The head-to-tail packing of the α_torque_ helices in the upper subring generates a periodic distribution of positive-to-negative charges along the CCW direction on the upper surface of the C ring (Supplementary information, Fig. S6f).

### The inter-subunit interactions of the C ring in the CCW state

**Fig. 3:**
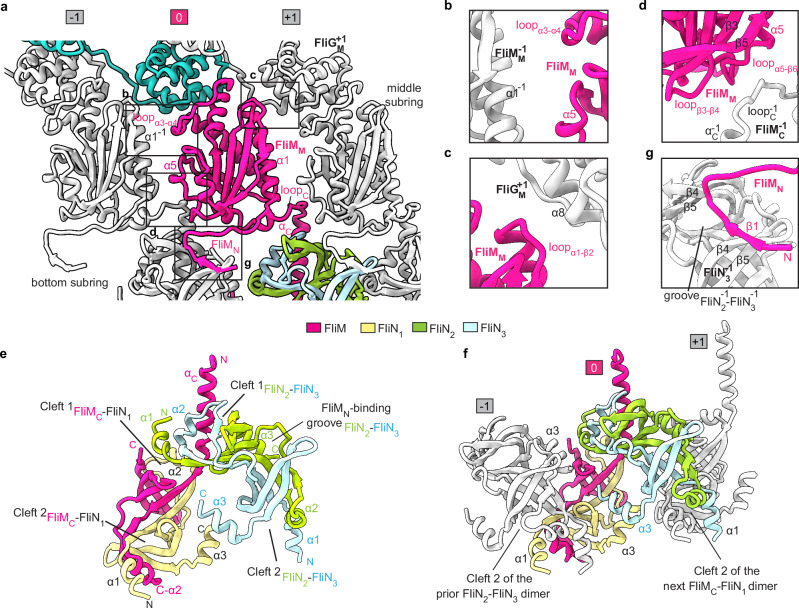
The inter-subunit interactions of the C ring in the CCW state. The inter-protomer interactions mediated by FliM_M_ in the CCW-C ring. Neighboring FliM_M_, FliM_N_, and FliG_M_ domains are colored in gray and numbered sequentially (**a**). **b**–**d** Detailed interactions of the FliM_M_ with neighboring FliM_M_^–1^ (**b**), FliG_M_^+1^ (**c**) and FliM_C_^–1^ (**d**) domains are illustrated as indicated. **e** Side view of the FliM_C_–FliN tetramer in the CCW-C ring. **f** The spiral structure of the bottom subring of the CCW-C ring and the α1-swapping interactions through Cleft 2 of the FliM–FliN tetramers between the protomers. **g** Interactions of FliM_N_ with the FliN_2_–FliN_3_ dimer of the prior protomer. The FliM, FliN_1_, FliN_2_ and FliN_3_ subunits of the focused protomer are colored in red, wheat, green and light blue in **a,** **e** and **f**, respectively. The FliM and its interacting domains are colored in red and gray in **b**–**d** and **g**, respectively.

The FliM and FliN subunits are extensively involved in the intra- and inter-protomer interactions in the C ring. FliM_M_ interacts with the prior and next FliM_M_ domains in the middle subring in a “shoulder-to-shoulder” manner via α5, loop_α3–α4_, α1, α_C_, and loop_C_ (Fig. 3a, b). The interfaces generate the relatively weak interactions and leave small gaps between the FliM_M_ domains (Fig. 3a). FliM_M_ also contacts α8 of the FliG_M_^+1^ domain of the next protomer via loop_α1–β2_ (Fig. 3a, c) and interacts with α_C_ and loop_C_ of the prior FliM_C_ domain to strengthen the middle subring and bridge the middle and bottom subrings, respectively (Fig. 3a, d).

In the bottom subring, FliM_C_ forms a heterodimer with the FliN_1_ subunit in a head-to-tail manner (Supplementary information, Fig. S6g), while the FliN_2_ and FliN_3_ subunits form a homodimer in the same fashion (Supplementary information, Fig. S6h). The dimerization generates two symmetrical clefts (Cleft 1 and Cleft 2) at each end of the dimers (Supplementary information, Fig. S6g, h). The two dimers undergo further dimerization and form a tetrameric structure through swapping the binding of α_C_ of FliM_C_ and α1 of FliN_2_ into Cleft 1 of each other in the protomer (Fig. 3e; Supplementary information, Fig. S6i). The tetramerization is also strengthened by the interactions between the α3 helices of FliN_1_ and FliN_3_ (Fig. 3e; Supplementary information, Fig. S6i). The α1 helix of FliN_1_ extends to bind into Cleft 2 of the FliN_2_–FliN_3_ dimer in the prior protomer, while the α1 of FliN_3_ extends to bind into Cleft 2 of the FliM_C_–FliN_1_ heterodimer in the next protomer (Fig. 3f). The α1-swapping interactions across Cleft 2 between the protomers generate a continuous spiral structure for the bottom subring with the FliM_C_–FliN_1_ dimer as the inner side and the FliN_2_–FliN_3_ dimer as the outer side (Fig. 3f; Supplementary information, Fig. S6j, k). The N-terminal region (residues 34–45) of FliM_N_ binds to the central groove in the prior FliN_2_–FliN_3_ dimer via the formation of an antiparallel β-sheet with β4 of FliN_3_^–1^ to lift up the outer side of the bottom subring (Fig. 3a, g).

### The structure of the C ring in the CW state

**Fig. 4:**
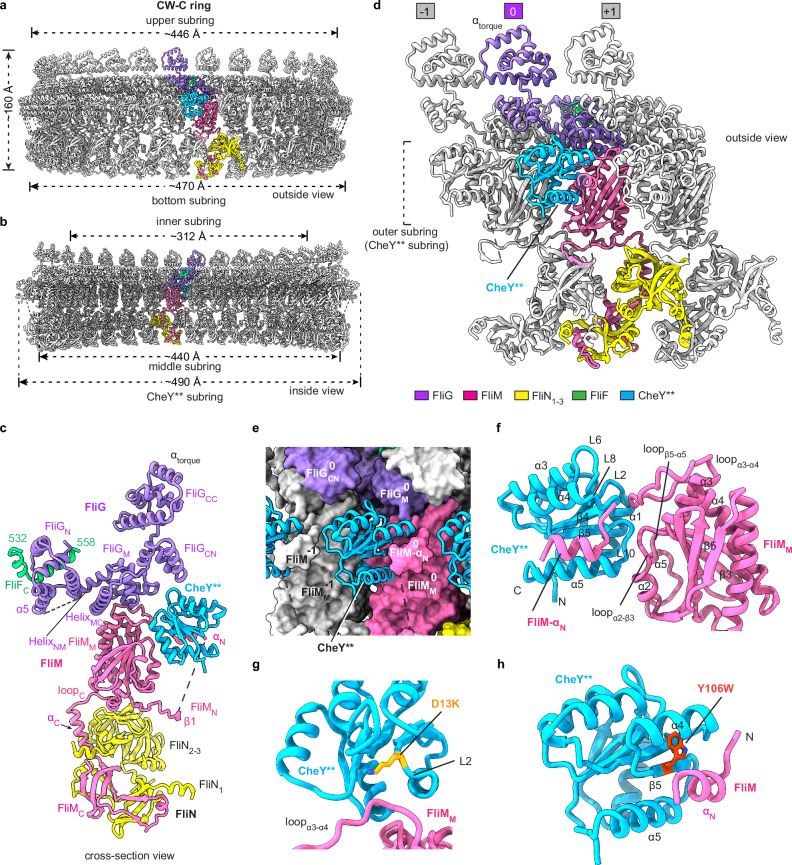
The structure of the C ring in the CW state. **a**, **b** Side (**a**) and cross-section (**b**) views of the structure of the C ring in the CheY**-induced CW state (CW-C ring). The subunits of FliG, FliM, FliN, FliF_C_ and CheY** in a protomer are colored in medium purple, pink, yellow, green and blue, respectively. **c** Cross-section view of the protomer of the CW-C ring. **d** Inter-protomer interactions of the CW-C ring. Neighboring protomers are colored in gray and numbered sequentially. **e** Surface representation of the interactions of CheY** with the FliG and FliM domains of the CW-C ring. CheY** is shown as cartoon. **f** Detailed interactions of CheY** with FliM_M_. **g**, **h** The interactions of the mutant residues D13K (**g**) and Y106W (**h**) of CheY** with FliM.

The CheY**-bound C ring in the CW state has a height of ~160 Å, which is the same as that of the C ring in the CCW state (Fig. 4a). However, the upper, middle and bottom subrings of the CW-C ring have diameters of ~446 Å, ~440 Å and ~470 Å, respectively (Fig. 4a, b), which are all smaller than those in the CCW-C ring, indicating that the binding of CheY** induces a more compressed structure of the C ring. The inner diameter of the inner subring is also shortened to ~312 Å (Fig. 4b). In the CW-C ring, FliG_C_ adopts a conformation that differs from that of the crystal structure of FliG_C_ in the mutant equivalent to the ^169^PAA^171^ deletion of FliG (Supplementary information, Fig. S7a).^22^ Structural superimposition revealed that related to FliG_M_, the FliG_N_ domain of FliG in the CW-C ring rotates by ~140° to interact with the helices α2 and α3 of the FliG_N_ domain of the next protomer (Supplementary information, Fig. S7b, c). CheY** is bound into an open groove between two adjacent protomers on the surface of the middle subring of the C ring and interacts with FliG_M_, FliM_M_ and the prior FliM_M_ domain (Fig. 4c–e). However, in contrast to previous predictions,^35^ CheY** has no interactions with FliN and the bottom subring in the structure.

The structure of CheY** in complex with the C ring is highly similar to those of the *Salmonella* beryllium fluoride (BeF_3_^–^)-activated CheY and of the CheY** alone and that in complex with a N-terminal portion of FliM from *Escherichia coli* (Supplementary information, Fig. S7d).^30,36^ At the interface with FliM, CheY** interacts with FliM_M_ and the N-terminal loop and the α_N_ helix of FliM_N_ (Fig. 4f). The α1 helix, L2, L6, L8 and L10 loops of CheY** form an open concave surface to interact with loop_α2–β3_, loop_α3–α4_, loop_β5–α5_ and α5 of FliM_M_ (Fig. 4f). CheY** binds to α_N_ and the N-terminal loop of FliM_N_ via the α4–β5–α5 surface (Fig. 4f). The mutated residue D13K of CheY** interacts with the loop_α3–α4_ of FliM_M_ (Fig. 4g), while the mutated residue Y106W is located on the α4–β5–α5 surface of CheY** and stacks with the α_N_ helix of FliM_N_ (Fig. 4h), suggesting that the double mutation activates CheY through generating direct interactions with the C ring. In the interactions with FliG, the L6 and L8 loops of CheY** clamp the α11 helix of FliG_M_ (Supplementary information, Fig. S7e). CheY** also contacts with the β2, β6 and β7 strands and the linking loop_β6–β7_ of FliM_M_^–1^ of the prior protomer via its α1–β2 surface (Supplementary information, Fig. S7f), which enhances the inter-protomer interactions in the middle subring.

### The CheY-induced conformational changes of the C ring

**Fig. 5:**
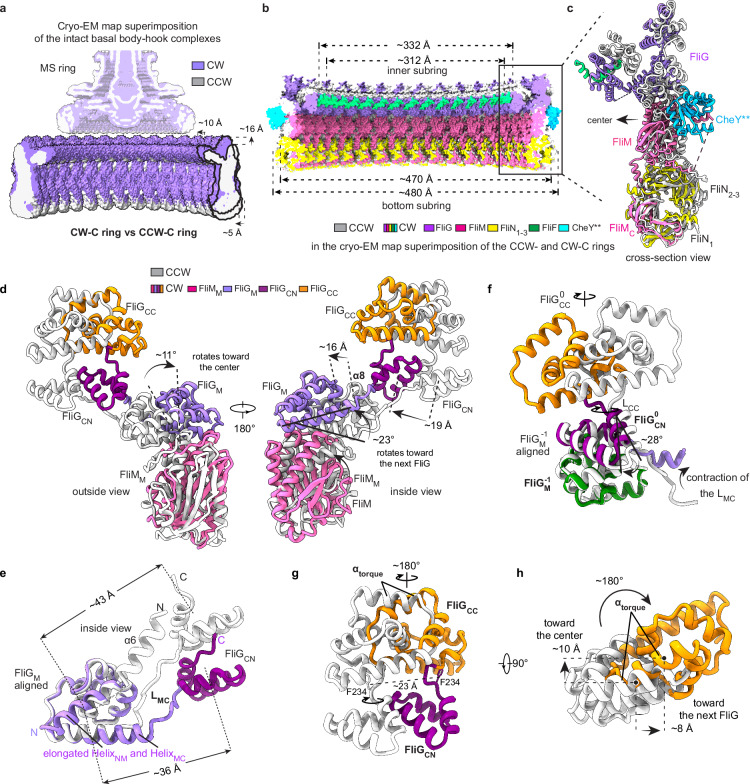
CheY**-induced conformational changes of the C ring in the intact motor. **a** Cryo-EM map superimposition of the C ring-containing basal body–hook complexes in the CCW and CW states. The density maps of the basal body–hook complexes in the CCW and CW states are colored in gray and medium purple, respectively. The changes are indicated by black arrows. **b** Structural comparison of the CCW-C and CW-C rings in the cryo-EM map superimposition of the CCW-C and CW-C rings in Supplementary information, Fig. S8b. **c** Structural comparison of the protomers of the CCW-C and CW-C rings in the structural superimposition of the C rings in (**b**). The protomers in the CCW-C and CW-C rings are colored in gray and as in Fig. 4a, respectively. **d** Structural comparison of the FliG_M_–FliG_C_–FliM_M_ domains in the structural superimposition of the CCW-C and CW-C rings. FliM_M_, FliG_M_, FliG_CN_ and FliG_CC_ from the CW-C ring are colored in pink, medium purple, purple and yellow, respectively. The domains in the CCW-C ring are colored in gray. Conformational changes are indicated by black arrows. **e** Structural comparison of the Helix_NM_ and Helix_MC_ helices from the CCW-C and CW-C rings through structural superimposition of FliG via the FliG_M_ domains. The elongated Helix_NM_ and Helix_MC_ in the FliG subunit are labeled as indicated. **f** Structural comparison of the FliG_CN_–FliG_CC_ domains in the CCW-C and CW-C rings by superimposing the FliG_M_^–1^ domains. **g**, **h** Side (**g**) and top (**h**) views of the conformational changes of the FliG_CC_ domain in the structural superimposition of the CCW-C and CW-C rings. The F234 residues are shown as sticks and labeled as indicated (**g**).

Structural and cryo-EM map superimpositions of the C ring-containing basal body–hook complexes in the CW and CCW states reveal that upon binding of CheY**, the whole structure of the C ring in the CW state undergoes a significant upward shift of ~16 Å towards the MS ring and the bacterial inner membrane (Fig. 5a; Supplementary information, Fig. S8a), which reduces the distance between the MS ring and C ring and shortens the overall length of the basal body–hook complex (Fig. 1c, f). Structural comparison of the CCW-C and CW-C rings revealed that, in contrast to the previous assumption of outward expansion of the C ring for the rotational switching,^12,25^ all four subrings of the CW-C ring undergo significant inward contractions towards the center of the motor upon binding of CheY**, resulting in smaller diameters of the four subrings (Fig. 5b; Supplementary information, Fig. S8b). The inward contraction of the inner FliG_N_–FliF_C_ subring decreases its inner diameter by ~20 Å, while the outer diameter of the bottom subring is shortened by ~10 Å (Fig. 5b).

In addition to the overall upward movement of the C ring in the motor, the binding of CheY** remodels the structures of the protomers in the CW-C ring (Fig. 5c). With the central β-strands of FliM_C_ as the fulcrum, FliM_M_ and α_C_ of FliM_C_ obliquely rotate by ~11° towards the center of the C ring, leading to the inward movement of the N-terminus of α_C_ of FliM_C_ by ~11 Å and of the FliG_M_-binding loop_α3–α4_ of FliM_M_ by ~21 Å (Supplementary information, Fig. S8c). The FliN_2_–FliN_3_ dimer also undergoes a rotation by ~11°, thereby retaining the inter-subunit interactions between FliM_C_ and the FliN subunits in the bottom subring (Supplementary information, Fig. S8d). In addition, FliM_M_ tilts by ~23° to the FliM_M_ domain of the next protomer (Supplementary information, Fig. S8e). The loop_C_ moves ~8 Å towards the center of the C ring and has a more extended conformation, thereby reducing the contacts of loop_C_ to the next FliM_M_ domain (Supplementary information, Fig. S8e). The N-terminal region of FliM_N_, which binds the FliN_2_–FliN_3_ dimer in the prior protomer, moves ~11 Å towards the center of the C ring (Supplementary information, Fig. S8f).

The binding of CheY** onto the C ring not only changes the orientations of all the domains of FliG, but also alters the distances between the domains in the protomer, leading to the overall structure of FliG in the CW state being distinct from that in the CCW state (Supplementary information, Fig. S8g). The packing of FliG_N_, FliG_M_ and FliG_C_ in FliG in the CW state is more compacted than that in the CCW state (Supplementary information, Fig. S8g). In accompany with the rotation of FliM_M_, FliG_M_ undergoes a rotation by ~11° towards the center of C ring and a tilt by ~ 23° towards the next protomer, leading to a movement by ~16 Å of the upper region of the α8 helix that is involved in the interactions with the FliG_CN_^+1^ domain of the next protomer (Fig. 5d). The FliG_CN_ domain also moves ~19 Å towards the center of the C ring (Fig. 5d). Despite the conformational changes, FliG_M_ in the CW state maintains an identical interface with FliM_M_ as that in the CCW state (Fig. 5d). FliG_N_ moves downwards by ~25 Å and rotates by ~180° towards the prior protomer (Supplementary information, Fig. S8h), resulting in the reduced diameter of the inner subring (Fig. 5b). The conformational changes of FliG_N_ induce the fusion of the α6 helix of FliG_N_ with the N-terminus of the Helix_NM_ helix of FliG_M_, which generates a longer helical structure for Helix_NM_ of FliG_M_ in the CW-C ring (Fig. 5e).

### The conformational changes of FliG_CC_ for rotational switching

**Fig. 6:**
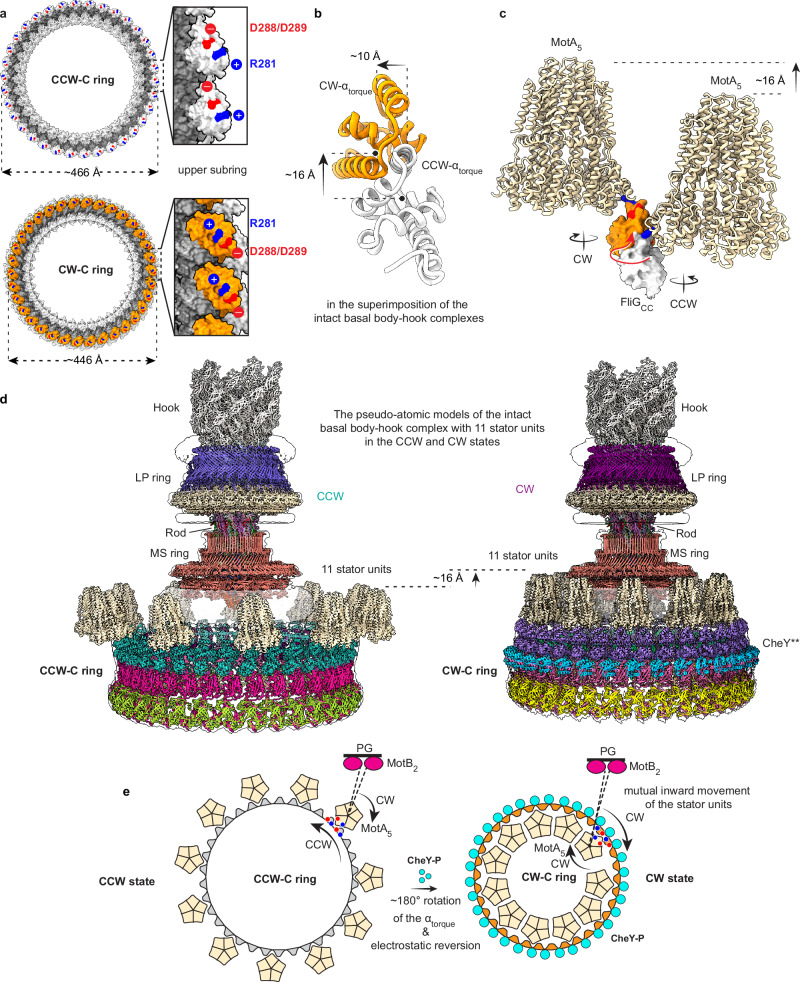
The proposed rotational switching mechanism of the bacterial flagellar motor. **a** Distribution of the charged residues R281, D288 and D289 on the upper surface of the CCW-C ring (upper) and the CW-C ring (bottom). The charged residues of the α_torque_ helices are colored in blue and red as positive and negative charges, respectively. The FliG_CC_ domains of the CCW-C and CW-C rings are colored in gray and yellow, respectively. **b** Cross-section view of the conformational changes of the FliG_CC_ domain in the cryo-EM map superposition of the basal body–hook complexes. In addition to the conformational changes illustrated in Fig. 5h, the FliG_CC_ domain shifts upward by ~16 Å in the motor in the CW state. **c** Side view of the relocation of a stator unit in the motor for the rotational switching. The structural models of the interactions of the MotA_5_ pentamer of a stator with FliG_CC_ were constructed according to the cryo-ET map of the *Helicobacter* motor in the CCW state,^37^ as shown as in Supplementary information, Fig. S10a. The stator units are colored in wheat and shown as cartoons. The FliG_CC_ domains are colored as in (**a**) and shown as surface. **d** The pseudo-atomic models of the intact basal body–hook complex with 11 stator units in the CCW (left) and CW (right) states. **e** Schematic diagram of the rotational switching mechanism of the flagellar motor. The binding of CheY** or CheY-P onto the C ring induces the upward and inward movements of the FliG_CC_ domains and reverses the orientations of the α_torque_ helices, which induces the relocation of the stator units in the bacterial inner membrane from the outer side (left) of the upper subring to the inner side (right) and triggers the rotation switching from CCW to CW.

The binding of CheY** also induces a significant contraction of the loop L_MC_ that connects FliG_M_ and FliG_CN_ and results in the formation of an elongated Helix_MC_, which not only reduces the distance between FliG_M_ and FliG_CN_ by ~7 Å (Fig. 5e), but also causes a CW rotation of FliG_CN_ by ~28° on the binding surface of the FliG_M_^–1^ domain in the prior protomer (Fig. 5f; Supplementary information, Fig. S9a). The rotation and movement of FliG_CN_ cause a rotation by ~180° and relocation of the hinge loop L_CC_ that connects FliG_CN_ and FliG_CC_ (Supplementary information, Fig. S9b). F234, the key residue stabilizing the orientation of FliG_CC_ in the protomer via hydrophobic interactions, in L_CC_ moves by ~23 Å and rotates by 180° (Fig. 5g; Supplementary information, Fig. S9b). As the consequence, the whole FliG_CC_ domain undergoes a rotation by ~180° and a movement by ~8 Å toward the next FliG_CC_ domain and inward contraction by ~10 Å to the center of the C ring (Fig. 5g, h; Supplementary information, Fig. S9c, d), which results in a reversed orientation of the α_torque_ helix and changes the periodic CCW distribution of the charged residues R281 and D288/D289 to a CW distribution on the upper surface of the CW-C ring (Fig. 6a). The changed distribution of the charged residues then reverses the surface electrostatic potential of the α_torque_ in the CW-C ring (Supplementary information, Figs. S6f, S9e). Thus, in the intact flagellar motor, the binding of CheY** onto the C ring finally induces an upward shift by ~16 Å, an inward movement by ~10 Å and an orientation reversion of α_torque_ to trigger the rotational direction switching of the motor from CCW to CW (Figs. 5g, h and 6b).

## Discussion

It is a long-standing question in the field about the rotational switching mechanism of the bacterial flagellar motor.^3,4,12,13^ The two cryo-EM structures of the *Salmonella* flagellar basal body–hook complexes in the CCW and CW states not only present the intact architectures of a flagellar motor, but also uncover the detailed structures and assembly of the CCW-C and CW-C rings. In contrast to the previously reported or proposed structures,^19^ FliG adopts a twisted “V”-shaped structure in the CCW-C ring and a more compacted structure in the CW-C ring. CheY** not only binds to FliM, but also interacts with FliG and the FliM^–1^ subunit in the prior protomer in the middle subring of the C ring. The binding of CheY** does not cause any outward expansion of FliG or the C ring. Instead, the C ring undergoes a notable upward movement toward the MS ring and the bacterial inner membrane, and inward contraction to its center. The CheY**-induced conformational changes of Helix_MC_ and L_MC_, and the subsequent structural changes of FliG_CN_ and L_CC_ eventually reverse the orientation of α_torque_ and electrostatic distribution on the upper surface of the C ring to trigger the rotational direction switching of the motor. The CheY**-induced upward movement and inward contraction of the C ring suggest that the stator units need to be relocated in the bacterial inner membrane through inward movements by an appropriate distance towards the center of the motor for the rotational switching.

A 24-Å cryo-ET map of the *Helicobacter* flagellar motor in the CCW state clearly shows that the stator units are bound onto the top surface of the C ring at the outer side of the upper subring (Supplementary information, Fig. S10a).^37^ The structural models of the protomer of the *Salmonella* CCW-C ring and of the MotA pentamer (MotA_5_) of a stator unit could be well matched to the densities of the *Helicobacter* C ring and the stator units, respectively, in the low-resolution cryo-ET map (Supplementary information, Fig. S10a).^37^ For each stator unit in the map, two adjacent FliG_CC_ domains of the C ring clamp the protruding MotA subunit from its two sides (Supplementary information, Fig. S10a). According to the interactions of the protomers with the stator unit, a pseudo-atomic structural model of the intact *Salmonella* flagellar motor with stator units in the CCW state was modeled (Fig. 6c, d; Supplementary information, Fig. S10b). The interactions of the protomers with a stator unit only allowed 11 stator units as the maximum number to be bound onto the C ring in the *Salmonella* motor, which is consistent with the previous biophysical analyses (Fig. 6d; Supplementary information, Fig. S10b).^38,39^ The structural model of the intact motor with the 11 stator units was perfectly matched to the ~69-Å cryo-ET map of the *Salmonella* flagellar motor in the CCW state applied with C11 symmetry (Supplementary information, Fig. S10c).^40^ The pseudo-atomic structural model of the *Salmonella* motor with the 11 stator units in the CW state was then built, according to the conformational changes of the FliG_CC_ domains (Fig. 6c, d; Supplementary information, Fig. S10b). The relocated stator units did not generate any structural collision and did not allow accommodation of more stator units on the C ring either (Fig. 6d; Supplementary information, Fig. S10b). We modeled the structure of the CW-C ring-containing basal body–hook complex of *S*. Typhimurium into the ~19-Å cryo-ET density map of the flagellar motor of *Vibrio alginolyticus* in a FliG-G215A mutant-based biased CW state (Supplementary information, Fig. S10d).^27^ The structure of the *Salmonella* C ring is well fitted with the cryo-ET density map of the *Vibrio* flagellar motor that also contains 34 protomers in the C ring (Supplementary information, Fig. S10d).^27^ The stator units in the CW state could be well accommodated in the notably protruding densities above the C ring in the inner membrane, which are closed to the inner side of the upper subring and were not annotated previously in the map (Supplementary information, Fig. S10d, e),^27^ suggesting that the stator units on the *Vibrio* motor indeed undergo inward relocation in the inner membrane.

The C ring plays a key role in the mechanosensing of the bacterial flagellar motor in response to mechanical stimuli in environments by mediating the loading of the stator units.^5,13,39,41^ The relatively separated FliG_N_–FliF_C_ subcomplex is connected to FliG_M_ via Helix_NM_ that tightly stacks with Helix_MC_ in the C ring. It is possible that the C ring adjusts the conformations of the FliG_CC_ domains via the Helix_NM_–Helix_MC_–FliG_CN_–FliG_CC_ module cascade upon conformational changes of the FliG_N_–FliF_C_ subcomplex and the MS ring under environmental stresses to load the stator units onto the motor (Supplementary information, Fig. S5g). The extensive inter-protomer interactions between the “Y”-shaped protomers in the C ring suggest that conformational changes of the protomers have synergistic effects on recruiting CheY-P for rotational switching (Supplementary information, Fig. S5g).^42^

It was assumed that the stator units were fixed in their positions during the rotational switching.^13,25^ The structures of the C ring-containing basal body–hook complexes and the structural modeling of the intact motors with the stator units in the low-resolution cryo-ET maps reveal that the stator units undergo a relocation adaptation process in accompany with the CheY-induced conformational changes of the C ring. In the CCW state, the stator units, which rotate in the CW direction, are located at the outer side of the upper subring of the C ring and trigger the rotation of the motor in the CCW direction. Upon binding of CheY-P, the stator units are relocated to the inner side of the upper subring of the C ring for adaptation with the reversed orientation of the α_torque_ helix of FliG_CC_, leading to the CW-direction rotation of the motor (Fig. 6e; Supplementary information, Fig. S10b and Videos S1, S2). For the relocation, the stator units likely keep the interactions with the FliG_CC_ domain and then undergo the rotation by 180° along with the rotation of the FliG_CC_ domains (as shown in Supplementary information, Video S2). There is also another possible manner, in which the stator units temporarily lose the interactions with FliG_CC_ and then directly move to the inner side of the upper subring of the C ring. In this relocation manner, the stator units move laterally in the inner membrane and do not undergo the rotation by 180°. Two recent studies also determined the structures of the C ring in the CCW and CW states.^43,44^ These studies also proposed the inward movement of the stator units from the outside to the inside of the C ring for the rotational switching. Different from our CW-C ring that was induced by CheY**, both studies obtained the CW-C ring that is locked by a FliG mutant with deletion of the residues ^169^PAA^171^. Due to the lack of the structures of the MS ring, rod, LP ring in the intact motor, both studies did not observe the upward shift of the C ring towards the MS ring upon CheY** binding for the rotational switching.^43,44^ Despite the diversities of the flagellar motors, the motor components of *S*. Typhimurium are highly conserved in bacterial species.^45^ The relocation of the stator units is possibly a general adaptation process for the rotational switching of the flagellar motors.

## Materials and methods

### Bacterial strains

The ∆*fliCD* strain of *Salmonella enterica* serovar Typhimurium LT2 (ATCC 700720) was generated in our previous study.^14^ The cells of *Salmonella* were grown in 2YT or Luria-Bertani (LB) broth media supplemented with 10 μg/mL streptomycin at 37 °C. The *Escherichia coli* BL21 (DE3) cells were grown in 2YT medium or LB supplemented with appropriate antibiotics at 37 °C.

### Purification of the intact basal body–hook complex in the default CCW state

The *∆fliCD* strain of *Salmonella enterica* serovar Typhimurium LT2 was initially cultured in 2YT medium supplemented with streptomycin (10 μg/mL) at 37 °C overnight. 1.6 mL of the cultured bacteria were transferred into 1 L of LB medium supplemented with streptomycin. After grown for 4.5 h at 37 °C, 12 L bacterial cells were harvested by centrifugation at 4000 rpm. The harvested cells were resuspended in 200 mL resuspension buffer consisting of 100 mM Tris-HCl, pH 8.0, 10 mM EDTA, 500 mM sucrose and 0.1 mg/mL lysozyme, and stirred over ice at 200 rpm on a shaker for 1 h. The cells were lysed by adding 1% (v/v) Triton X-100 and 10 mM MgSO_4_. After incubation for 10 min, the lysates were further lysed by adding 200 mL resuspension buffer pre-mixed with 1% (v/v) Triton X-100 and 10 mM MgSO_4_, and incubated at 4 °C for 1 h. The supernatant of the cell lysates was collected after removal of the cell debris through centrifugation at 14,000 rpm for 15 min. The pH value of the supernatant was then adjusted to 9.0 by adding NaOH. Denatured proteins in the supernatant were removed by low-speed centrifugation at 14,000 rpm for 10 min. The supernatant was collected and subjected to high-speed centrifugation at 35,000 rpm for 1 h. After centrifugation, the membrane pellets containing the flagellar basal body–hook complex were harvested and resuspended in 3 mL TET buffer consisting of 10 mM Tris-HCl, pH 8.0, 5 mM EDTA and 0.1% (v/v) Triton X-100. The samples were homogenized gently with a Dounce homogenizer (7 mL Tissue Grinder, Dounce, #357542) and incubated at 4 °C for 1 h. The supernatant of the homogenates was harvested after low-speed centrifugation at 12,000 rpm for 10 min. Following addition of 900 μL Percoll (Biosharp, #BS909), the sample was diluted by adding TET buffer to the final volume of 6 mL and loaded into the SW41 tubes for centrifugation at 10,000 rpm for 10 h at 4 °C. After density gradient centrifugation, the fractions containing the intact basal body–hook complex were collected by drop collection (500 μL per tube) and validated by SDS-PAGE and Coomassie blue staining. The fraction containing the basal body–hook complex was loaded into a Superose 6 10/300 GL column (GE Healthcare) pre-equilibrated with TET buffer. The eluted peaks were assessed by negative staining analyses. The fractions containing the intact basal body–hook complex were collected and concentrated using a Nanosep™ centrifugal tube (PALL, 300 kDa, #OD300C33), and immediately subjected to cryo-EM analysis.

### Expression and purification of the recombinant CheY** protein

The gene encoding the CheY protein was initially amplified from the genomic DNA of *Salmonella enterica* serovar Typhimurium LT2 using standard PCR method. The DNA fragment encoding the D13K/Y106W mutant of CheY (CheY**) was generated through site-directed mutagenesis PCR method and cloned into the pET-14b vector. A Twin-Strep-tag II tag was linked to the C-terminus of CheY** via an HRV3C protease cleavage site. The plasmid was verified by DNA sequencing. To express the recombinant CheY** protein, the *E. coli* BL21 (DE3) cells transformed with the pET-14b-CheY** plasmids were induced by 350 μM IPTG when the OD_600_ value of the cell culture reached a range of 0.6–0.8. After being cultured at 18 °C for 14 h, the cells were harvested through centrifugation at 4000 rpm for 30 min and resuspended in a buffer containing 20 mM Tris-HCl, pH 8.0, and 150 mM NaCl. The cells were then lysed using high-pressure homogenization. After removal of the cell debris by centrifugation at 18,000 rpm for 20 min, the supernatant of the cell lysates was collected and loaded onto a STarm Streptactin column (Smart-Lifesciences, #SA092100) pre-equilibrated with the resuspension buffer. The CheY** proteins were eluted with the buffer consisting of 20 mM Tris-HCl, pH 8.0, 150 mM NaCl, and 2.5 mM D-desthiobiotin. After PreScission protease digestion, the sample was loaded onto a Superdex 75 increase 10/300 GL column (GE Healthcare) pre-equilibrated with the resuspension buffer. The eluted fractions containing the CheY** protein were collected and validated by SDS-PAGE and Coomassie blue staining, and concentrated to a final concentration of 2 mg/mL.

### Reconstitution of the basal body–hook complex in the CW state

To obtain the particles of the flagellar basal body–hook complex in the CW state, 5 μL of the flagellar basal body–hook complex in the CCW state was mixed with 1 μL of CheY** protein (2 mg/mL). The sample was incubated at room temperature for 15 min and then on ice for 1.5 h before vitrification for cryo-EM analysis.

### Negative staining analysis

For negative staining, 2.5 μL of protein samples were loaded onto a glow-discharged carbon film supported copper grid (200 mesh) at room temperature. After 2 min incubation, the solution on the grid was removed by the filter paper. The samples were then stained with 5 μL of 3% uranyl acetate for three times (for 10 s at the first and second times, and for 1 min at the third time). After removal of the uranyl acetate solution, the grids were air-dried at room temperature for 30 min. The prepared grids were examined using a transmission electron microscope operated at a voltage of 80 kV (HT7700, HITACHI).

### Cryo-EM sample preparation

Vitrification experiments were conducted using an FEI Vitrobot Mark IV. To vitrify the flagellar basal body–hook complex in the CCW state, 3 μL of the samples were applied to the holey carbon grids (Quantifoil, R 0.6/1, 300 mesh, Au) and the ultra-thin carbon film supported grids (Quantifoil, thickness of 2 nm, R 2/1, 200 mesh, Cu), which were glow-discharged before use. After sample loading, the holey carbon grids were blotted for 2 s at 100% humidity and 4 °C. The blotting force was set as –5. The waiting and draining time were 5 s and 2 s, respectively. The ultra-thin carbon film supported grids were blotted for 2 s with the blotting force of –15, the waiting time of 60 s at 100% humidity and 4 °C. To vitrify the CheY**-bound basal body–hook complex in the CW state, 3 μL of the samples were loaded onto a glow-discharged holey carbon grid (Quantifoil, R 0.6/1, 200 mesh, Au). The grids were blotted for 4 s with the blotting force of 10, waiting time of 30 s and draining time of 2 s at 100% humidity and 4 °C. After blotting, the grids were immediately immersed into the liquid ethane cooled by liquid nitrogen via plunge-freezing. All cryo-EM grids were stored in liquid nitrogen for subsequent data collection.

### Cryo-EM data collection

Cryo-EM images of the basal body–hook complex in the CCW state on the holey carbon grids were collected on an FEI Titan Krios electron microscope operated at 300 kV with a Gatan K3 camera at a nominal magnification of 81,000 using the EPU software (Thermo Fisher Scientific). A total of 6283 movies were acquired with defocus values ranging from 1.5 μm to 1.8 μm. The total dose of the electron beam was set to ~50 electrons/pixel with a pixel size of 1.1 Å/pixel, resulting in a total exposure dose of ~41 e^–^/Å^2^. Cryo-EM images of the basal body–hook complex in the CCW state on the ultra-thin carbon film supported grids were collected using an FEI Titan Krios electron microscope equipped with a Gatan K2 summit camera and operated at 300 kV. A total of 6169 movies were collected in a super-resolution mode at a nominal magnification of 105,000 using the serialEM software. The defocus values were set from 1.0 μm to 2.5 μm. The pixel size was set to 0.666 Å/pixel. The dose rate of the electron beam was set to ~8 e^–^/s per physical pixel, and the frame rate was set to 5 frames/s and the exposure time was set to 10 s, resulting in a total exposure dose of ~45 e^–^/Å^2^. For the data collection of the CheY**-bound basal body–hook complex in the CW state, a total of 15,225 electron-event representation (EER) movies were acquired on an FEI Titan Krios electron microscope equipped with a Falcon 4 camera via the EPU software. The microscope was operated at 300 kV with a nominal magnification of 105,000. Defocus values were set in the range of 1.2 μm to 1.8 μm. The pixel size was set to 1.2 Å/pixel and the exposure time was set to 7.24 s, resulting in a total exposure dose of ~50 e^–^/Å^2^.

### Cryo-EM data processing

For the reconstruction of the basal body–hook complex in the CCW state, 6283 and 6169 cryo-EM images obtained from the holey carbon and the ultra-thin carbon film supported grids, respectively, were individually imported into the RELION.^46^ After motion-correction using MotionCor2,^47^ the dose-weighted micrographs were individually imported into the cryoSPARC.^48^ Contrast Transfer Function (CTF) estimation was performed using the patch CTF estimation. The dataset collected from the holey carbon grids was used to reconstruct the density map of the C ring in the CCW state. The particles containing a highly ordered C ring were manually picked for analysis. A total of 19,627 particles centered on the CCW-C ring were extracted with a box size of 600 × 600. The particles were then subjected to 2D classification. The 2D class average clearly revealed the C34 symmetry of the C ring from the top-down view. There was no other symmetry variation observed. After ab initio 3D reconstruction, heterogeneous refinements were performed with the C32–C36 volumes from the non-uniform refinement as references for analysis of the symmetry heterogeneity. However, further 2D classification did not yield any top-down 2D class averages of the C ring in other symmetries, indicating the predominance of the C34 symmetry of the C ring in the dataset. To obtain a high-resolution structure, the C34 symmetry was applied to reconstruct the CCW-C ring structure with the 19,627 particles. After homogeneous refinement and local refinement with the C ring mask and C34 symmetry on the volume obtained in the non-uniform refinement, a density map of the CCW-C ring was reconstituted to an average resolution of 5.9 Å. After symmetry expansion and focused refinement on the four protomers of the CCW-C ring using the C1 symmetry, the resolution was further improved to 4.0 Å. The particles were then re-centered on the MS ring and re-extracted to reconstruct the intact basal body–hook complex containing the CCW-C ring. With a box size of 800 × 800, a total of 10,318 particles of the intact basal body–hook complex were obtained. After 2D classification, ab initio 3D reconstruction, and non-uniform refinement with the C34 symmetry, a density map of the intact C ring-containing basal body–hook complex in the CCW state was reconstructed to a resolution of 7.4 Å.

The dataset collected from the ultra-thin carbon film supported grids was used to determine the high-resolution density maps of the membrane-anchored part, including the rod, export apparatus, LP ring, MS ring, and hook of the basal body–hook complex in the CCW state. A total of 22,192 particles centered on the MS ring with an extraction box size of 512 × 512 were obtained by manual picking. A subset of 20,964 particles of the membrane-anchored part was then subjected to ab initio 3D reconstruction. After 3D refinement, a density map of the membrane-anchored part was obtained with a resolution of 4.1 Å. Further local refinements with different masks produced six locally refined maps of the membrane-anchored part, including the LP ring (with C26 symmetry), the whole rod with the export apparatus and the part of the hook, the distal rod with the part of the hook, the proximal rod with the export apparatus, the MS ring with the proximal rod and the export apparatus, and the β-collar–RBM3 subrings of the MS ring (with C34 symmetry), at the resolutions of 3.0 Å, 3.8 Å, 3.8 Å, 3.7 Å, 4.3 Å and 3.2 Å, respectively.

To reconstruct the cryo-EM map of the CheY**-bound C ring in the CW state, 15,225 EER movies were motion-corrected in cryoSPARC, followed by patch CTF estimation. The particles containing a highly ordered C ring were manually picked. A total of 46,058 particles centered on the CW-C ring were obtained and extracted with a box size of 600 × 600. The particles were then subjected to 2D classification. The 2D class average also revealed the C34 symmetry of the CW-C ring from the top-down view. There was not any other symmetry variation observed. After ab initio 3D reconstruction and 3D classification using RELION, a subset of 31,073 particles with good shapes of the membrane-anchored part and the C ring were selected. A round of 2D classification was further carried out in cryoSPARC. After ab initio 3D reconstruction, heterogeneous refinements were performed with the C32–C36 volumes from the non-uniform refinement as references for analysis of the symmetry heterogeneity. Further 2D classification did not yield top-down 2D class averages of the CW-C ring in other asymmetries, indicating the predominance of the C34 symmetry of the CheY**-bound CW-C ring in the dataset. To solve a high-resolution structure of the CW-C ring, the C34 symmetry was applied to the reconstruction of the CW-C ring. To reconstruct the CW-C ring, the membrane-anchored part was subtracted from the particles obtained in the homogeneous refinement following the non-uniform refinement that produced the C34-volume reference of the C ring. Further 2D classification confirmed the C34 symmetry of the CW-C ring in these particles. After ab initio 3D reconstruction with non-uniform refinement and local refinement with the C ring mask and C34 symmetry, a density map of the CW-C ring with an average resolution of 5.6 Å was obtained. After symmetry expansion and focused refinement on the four protomers of the CW-C ring using the C1 symmetry, the resolution was further improved to 4.4 Å. The particles obtained in RELION were then re-centered on the MS ring and re-extracted to reconstruct the intact motor–hook complex containing the CW-C ring. With a box size of 800 × 800, a total of 27,939 particles of the intact basal body–hook complex were obtained after 2D classification. After ab initio 3D reconstruction, non-uniform refinement and homogenous refinement with C34 symmetry, a density map of the intact C ring-containing basal body–hook complex in the CW state was reconstructed to the resolution of 4.3 Å. The particles from the homogeneous refinement were re-centered on the MS ring and re-extracted with a box size of 512 × 512. After 2D classification, a subset of 24,191 particles was selected. After ab initio 3D reconstruction and non-uniform refinement, the membrane-anchored part was reconstructed to a resolution of 3.4 Å. Further local refinements with different masks produced density maps, including the LP ring (with C26 symmetry), the whole rod with the export apparatus and the part of the hook, the distal rod with the part of the hook, the proximal rod with the export apparatus, the MS ring with the proximal rod and the export apparatus, the β-collar–RBM3 subrings of the MS ring (with C34 symmetry) and the β-collar–RBM3 subrings of the MS ring with the proximal rod, at the resolutions of 2.8 Å, 3.3 Å, 3.3 Å, 3.3 Å, 3.8 Å, 2.9 Å and 3.7 Å, respectively.

All map sharpening was conducted automatically in cryoSPARC. The protomer density maps of the CCW-C and CW-C rings were further sharpened using DeepEMhancer.^49^ The Fourier Shell Correlation (FSC) curves of the reconstructions were generated by cryoSPARC. The local resolution maps were calculated in cryoSPARC using the two independent half-maps of each reconstruction as input and further visualized in Chimera.^50^ Average resolutions were estimated based on the corrected FSC curves with an FSC threshold of 0.143. All masks used for the local refinement in this study were generated in Chimera and ChimeraX.^51^ Particles were formatted using the UCSF PYEM (10.5281/zenodo.3576630). Structure figures were prepared in Chimera, ChimeraX and PyMol (https://pymol.org/2/). Density map superimpositions and structural model superimpositions were performed in Chimera, ChimeraX, and PyMol.

### Model building and refinement

Initial model building of the C ring protomer was carried out using the previously reported crystal structures of the subdomains of FliG, FliF, FliM, and FliN from *Aquifex aeolicus*, *Thermotoga maritima* and *Salmonella* Typhimurium (PDB: 3HJL,^19^ 4FHR,^24^ 5TDY,^20^ 4YXB^23^ and 4YX1^23^), respectively, as reference models with assistance of AlphaFold2^52^ in the deepEMhancer-sharpened 4.0-Å density map of the CCW-C ring protomers. To build the model of the protomer, the domains and subcomplexes of FliG, FliM and FliN of a protomer, including the FliG_M_, FliG_CN_, FliG_CC_ and FliM_NM_ domains, and the FliG_N_–FliF_C_, and FliM_C_–FliN_1–3_ subcomplexes, were respectively fitted into the map using ChimeraX and rigidly refined using Phenix.^53^ The residues of FliM_M_ were manually modeled according to their clear densities in Coot.^54^ The linking regions between FliM_M_ and FliM_C_, FliG_M_ and FliG_CN_, FliG_α4–α5_ and FliG_CN_–FliG_CC_ were manually built in Coot based on the volume. The resulting protomer model was further rigidly refined in both the cryoSPARC-auto sharpened and the deepEMhancer-sharpened maps of the four protomers in real space using Phenix. The 34-fold structural model of the CCW-C ring was obtained by applying the C34 symmetry to the model of the protomer in the 5.9-Å density map in Chimera and further rigid-body refinement in Phenix. The final CC_box_ and CC_mask_ values of the CCW-C ring after refinement are 0.80 and 0.58, respectively. To build the model of the C ring in the CW state, the structural model of CheY** was initially generated using the crystal structure of CheY (PDB: 2FLW^36^). The refined structures of the protomers of the CCW-C ring were used for structural modeling of the protomer in the deepEMhancer-sharpened 4.4-Å density map of the CW-C ring protomers. Domains of CheY**, FliG, FliM and FliN of a protomer were first fitted into the map and rigidly refined in real space using Phenix. The linking region of the refined domains were manually built in Coot. The residues of the FliM_M_ domain were manually modeled according to their clear density in the map. The resulting protomer model was further refined in both the cryoSPARC-auto sharpened and the deepEMhancer-sharpened maps of the protomers in real space using Phenix. The final model of the CW-C ring was obtained by applying the C34 symmetry with the CW protomer in the 5.6-Å density map of the CW-C ring in Chimera and subsequent rigid-body refinement in Phenix. The final CC_box_ and CC_mask_ values of the CW-C ring after refinement are 0.77 and 0.52, respectively.

In the motor containing the CW-C ring, the structural model of the LP ring was built using the previously determined LP ring structure (PDB: 7CBL^14^). Other models, including the distal rod–hook complex, the proximal rod with the export apparatus and 11 FliF loops, the β-collar–RBM3 subrings of the MS ring with the Dc loops of FlgB and the α1 helices of FliE, were generated by ModelAngelo^55^ in their respective locally refined density maps. These models were manually built in Coot and refined in real space in Phenix. The inner RBM2 subring of the MS ring was built from the reported cryo-EM structures (PDB: 7CGO),^14^ while the outer RBM1–RBM2 subrings were built from the RBM1–RBM2 protomer generated by AlphaFold2. Eight of the 11 protomers of the outer RBM1–RBM2 subrings were included and refined in the map using Phenix. The whole structure of the membrane-anchored part of the basal body–hook complex in the CW state was generated by integrating the above refined models into the 3.4-Å density map and then rigidly refined as a whole body in Phenix. The final structural model of the intact C ring-containing basal body–hook complex in the CW state was obtained by integrating the refined models of the membrane-anchored part and the CW-C ring into the 4.3-Å density map of the basal body–hook complex in the CW state. The membrane-anchored part of the CCW-C ring-containing motor was built from the refined models of the CW-C ring-containing motor and refined in the corresponding locally refined maps and then integrated into the 4.1-Å density map. The final model of the intact basal body–hook complex containing the CCW-C ring was obtained by combining the membrane-anchored part and the CCW-C ring into the 7.4-Å density map.

All final models were validated using MolProbity.^56^ Root mean square deviation (RMSD) values and the electrostatic distributions were calculated using PyMol. The model resolutions were estimated by phenix.mtriage using the model-based noise-free and experimental maps with an FSC criterion of 0.5. Angle and distance measurements were performed in ChimeraX. The statistics of data collection, data processing, model building, refinement and validation related to the basal body–hook complex in the CCW and CW states are listed in Supplementary information, Tables S1, S2, respectively.

## Supplementary information

Supplementary information, Figure S1

Supplementary information, Figure S2

Supplementary information, Figure S3

Supplementary information, Figure S4

Supplementary information, Figure S5

Supplementary information, Figure S6

Supplementary information, Figure S7

Supplementary information, Figure S8

Supplementary information, Figure S9

Supplementary information, Figure S10

Supplementary information, Table S1

Supplementary information, Table S2

Supplementary information, Video S1 The CheY-induced conformational changes of the C ring protomer

Supplementary information, Video S2 The CheY-induced conformational changes of the whole C ring and the relocation of the stator units for the motor’s rotational switching from CCW to CW

## Data Availability

Cryo-EM density maps and coordinates related to the flagellar basal body–hook complex in the CCW state have been deposited in EMDB and PDB under the following accession codes: the flagellar C ring-containing basal body–hook complex (EMD-37679, PDB: 8WO5); the protomers of the CCW-C ring (EMD-38546, PDB: 8XP0); the CCW-C ring with C34 symmetry (EMD-39349, PDB: 8YJT); the membrane-anchored part, including the rod, export apparatus, LP ring, MS ring and hook, of the basal body–hook complex in the CCW state (EMD-37630, PDB: 8WLT); the LP ring with C26 symmetry (EMD-37618, PDB: 8WLE); the whole MS ring with the proximal rod and export apparatus (EMD-37625, PDB: 8WLN); the whole rod with the export apparatus and the part of the hook (EMD-37628, PDB: 8WLQ); the distal rod with the part of the hook (EMD-37627, PDB: 8WLP); the proximal rod with the export apparatus and 11 FliF loops (EMD-37619, PDB: 8WLH) and the β-collar–RBM3 subrings of the MS ring with C34 symmetry (EMD-37620, PDB: 8WLI). Cryo-EM density maps and coordinates related to the flagellar basal body–hook complex in the CW state have been deposited in EMDB and PDB under the following accession codes: the intact CheY**-bound basal body–hook complex (EMD-37684, PDB: 8WOE); the protomers of the CW-C ring (EMD-38547, PDB: 8XP1); the CheY**-bound CW-C ring with C34 symmetry (EMD-37570, PDB: 8WIW); the membrane-anchored part, including the rod, export apparatus, LP ring, MS ring and hook, of the basal body–hook complex in the CW state (EMD-37611, PDB: 8WL2), the LP ring with C26 symmetry (EMD-37547, PDB: 8WHT); the whole MS ring with the proximal rod and export apparatus (EMD-37605, PDB: 8WKQ), the whole rod with the export apparatus and the part of the hook (EMD-37601, PDB: 8WKK); the distal rod with the part of the hook (EMD-37600, PDB: 8WKI); the proximal rod with the export apparatus and 11 FliF loops (EMD-37594, PDB: 8WK3); the β-collar–RBM3 subrings of the MS ring with C34 symmetry (EMD-37590, PDB: 8WJR) and the β-collar–RBM3 subrings of the MS ring with the proximal rod (EMD-37595, PDB: 8WK4).
