# Supplementary material for: Structural basis of the bacterial flagellar motor rotational switching
Source: Cell Res. 2024 Aug 23;34(11):788–801. doi: 10.1038/s41422-024-01017-z (PMC11528121; doi:10.1038/s41422-024-01017-z)
Supplement: Supplementary file 2 — Supplementary information, Figure S2 [file 41422_2024_1017_MOESM2_ESM.pdf]

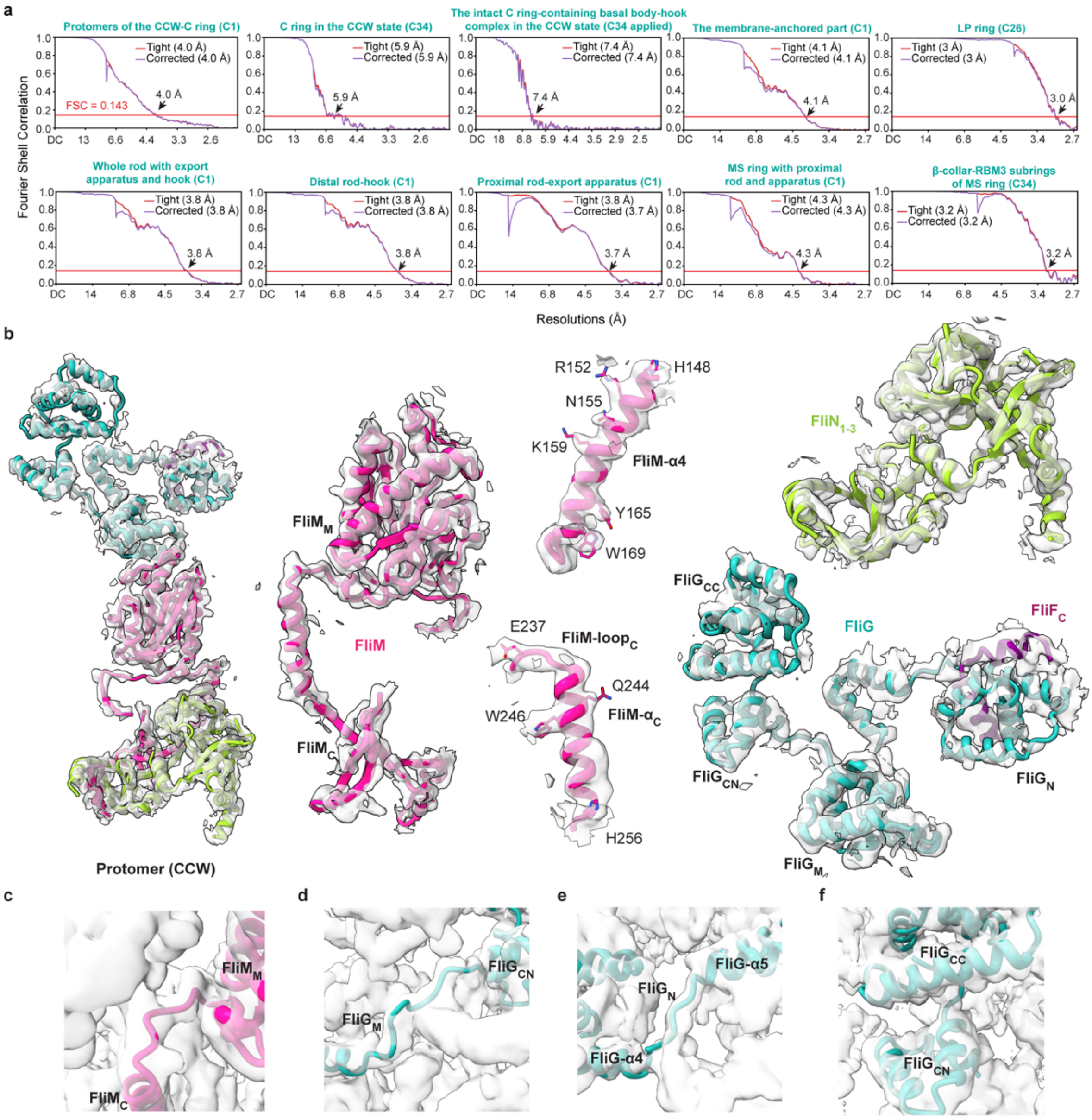

Supplementary information, Figure S2. FSC curves and representative cryo-EM density maps of the components of the CCW-C ring.

a, The Fourier Shell Correlation (FSC) curves for the reconstructions of the basal body-hook complex in the CCW state. The tight and corrected FSC curves of the reconstructions are illustrated. The average resolutions are estimated based on the corrected FSC curves at the FSC=0.143 criterion.

b-f, Representative cryo-EM density maps of the protomer, the FliG, FliM and FliN subunits of the CCW-C ring. Representative residues of the $FliM_M$ domain are shown as sticks (**b**). Represent models and densities of the linking regions between domains in the C ring after refinement are illustrated (**c-f**).
