# Supplementary material for: Structural basis of the bacterial flagellar motor rotational switching
Source: Cell Res. 2024 Aug 23;34(11):788–801. doi: 10.1038/s41422-024-01017-z (PMC11528121; doi:10.1038/s41422-024-01017-z)
Supplement: Supplementary file 3 — Supplementary information, Figure S3 [file 41422_2024_1017_MOESM3_ESM.pdf]

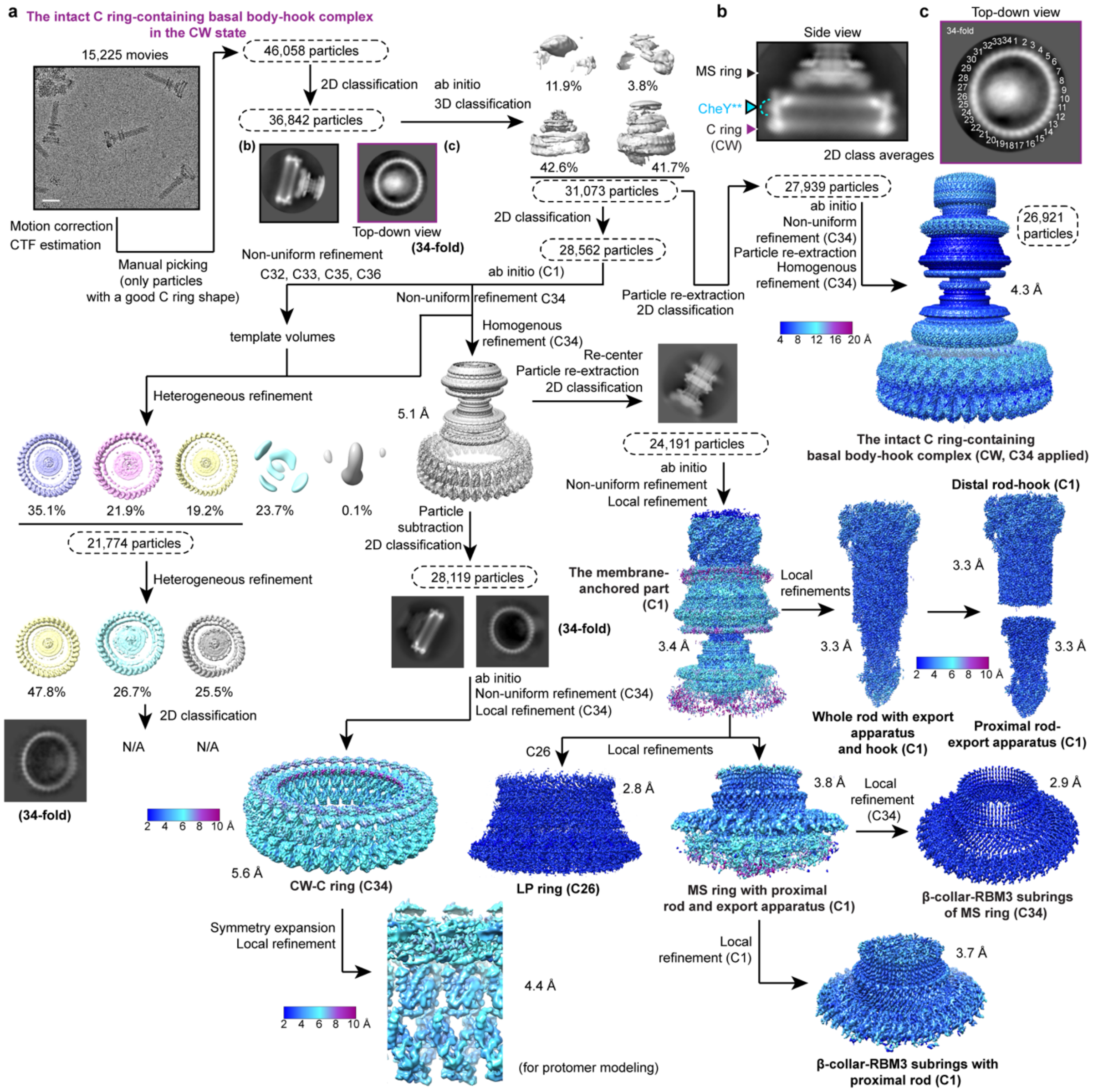

Supplementary information, Figure S3. Cryo-EM data processing of the flagellar basal body-hook complex in the CW state.

a, The flow chart for the cryo-EM data processing of the C ring-containing basal body-hook complex in the CW state. The local resolution maps were calculated in cryoSPARC using two independent half maps of each reconstruction as input. All density maps were prepared using *Chimera* and *ChimeraX*. Scale bar for the cryo-EM micrographs, 50 nm. N/A, not available.

b, A representative 2D class average of the C ring in the CW state with the MS ring from the side view.

c, A representative 2D class average of the CW-C ring from the top-down view.
