# Supplementary material for: Structural basis of the bacterial flagellar motor rotational switching
Source: Cell Res. 2024 Aug 23;34(11):788–801. doi: 10.1038/s41422-024-01017-z (PMC11528121; doi:10.1038/s41422-024-01017-z)
Supplement: Supplementary file 4 — Supplementary information, Figure S4 [file 41422_2024_1017_MOESM4_ESM.pdf]

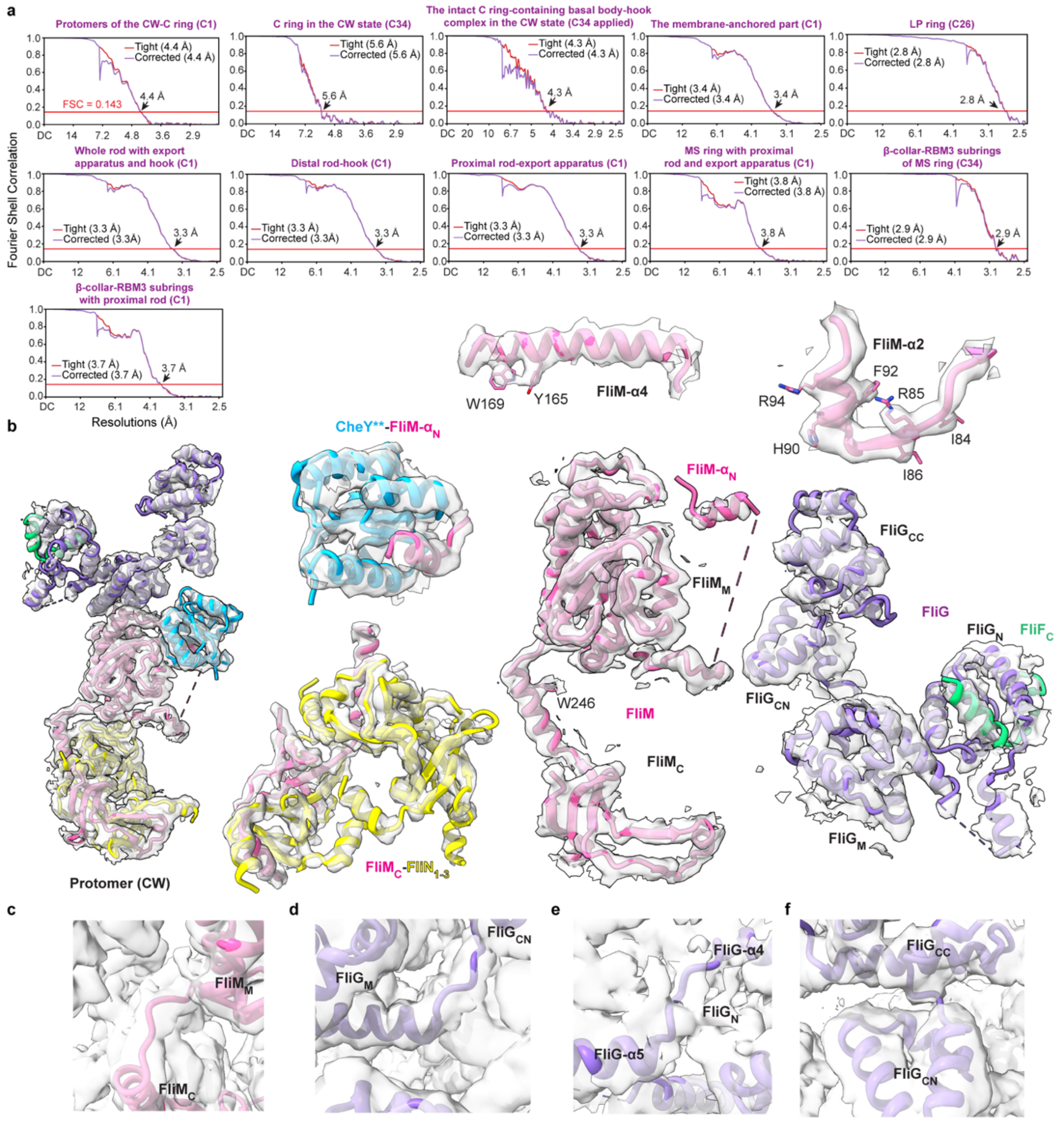

Supplementary information, Figure S4. FSC curves and representative cryo-EM density maps of the components of the CW-C ring.

a, The FSC curves for the reconstructions of the basal body-hook complex in the CW state. The tight and corrected FSC curves of the reconstructions are illustrated. The average resolutions are estimated based on the corrected FSC curves at the FSC=0.143 criterion.

b-f, Representative cryo-EM density maps of the protomer, the FliG, FliM and FliN subunits of the CW-C ring. Representative residues of the FliM$_M$ domain are shown as sticks (**b**). Represent models and densities of the linking regions between domains in the C ring after refinement are illustrated (**c-f**).
