# Supplementary material for: Structural basis of the bacterial flagellar motor rotational switching
Source: Cell Res. 2024 Aug 23;34(11):788–801. doi: 10.1038/s41422-024-01017-z (PMC11528121; doi:10.1038/s41422-024-01017-z)
Supplement: Supplementary file 5 — Supplementary information, Figure S5 [file 41422_2024_1017_MOESM5_ESM.pdf]

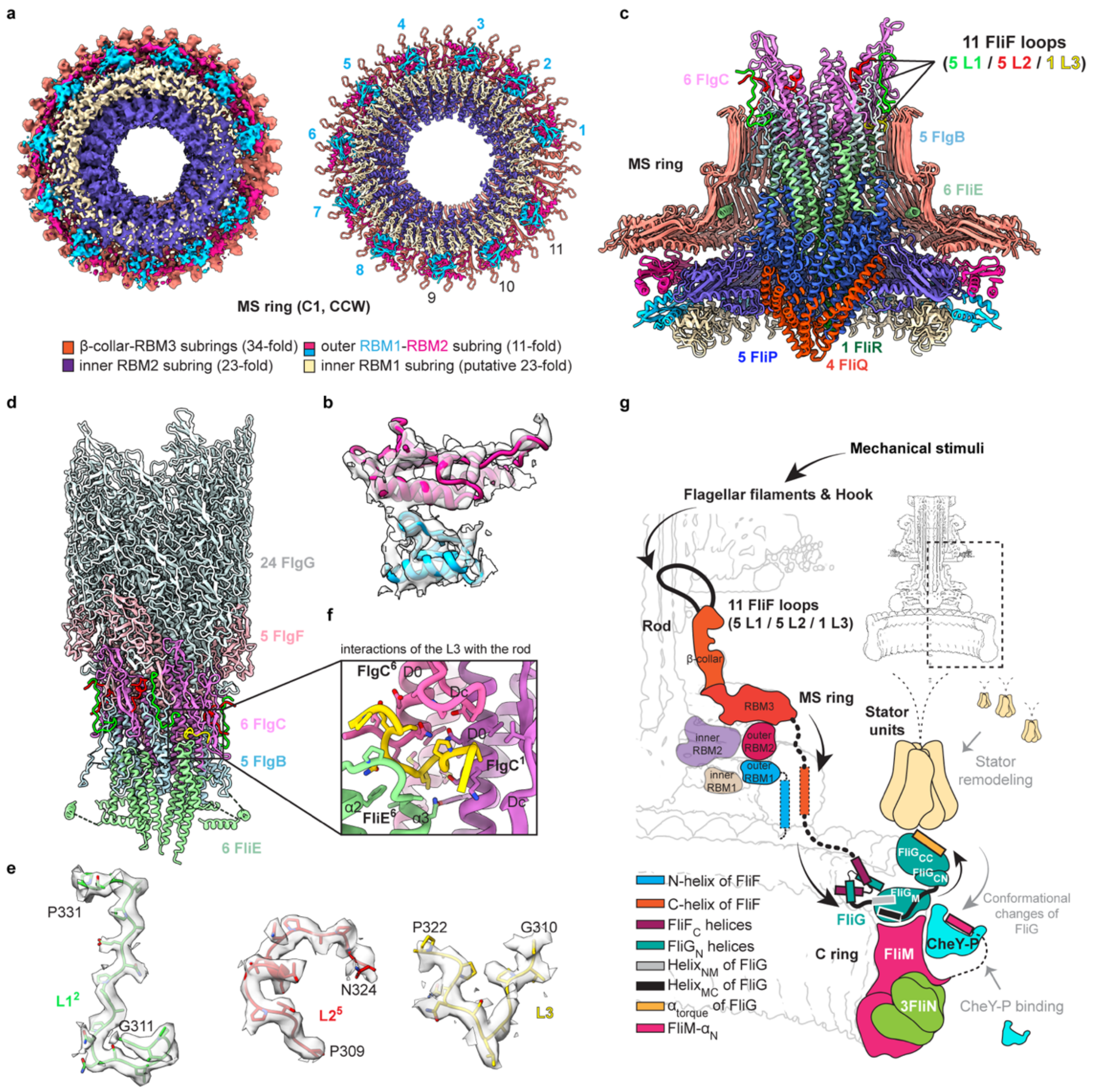

Supplementary information, Figure S5. Schematic diagram for potential mechanosensing mechanism of the flagellar motor, and interactions of the MS ring with the rod in the C ring-containing motors.

a, Bottom views of the cryo-EM density map (left) and structure (right) of the MS ring from the basal body-hook complexe in the CCW state. The 4.3-Å density map of the MS ring was reconstructed with C1 symmetry as shown in Supplementary information, Fig. S1a is illustrated. The 34-fold β-collar-RBM3, 23-fold inner RBM2, 11-fold outer RBM2, 11-fold outer RBM1 and putative 23-fold inner RBM1 subrings are colored in salmon, purple, red, blue and wheat, respectively. In this model, the putative 23-fold RBM1 subring and 3 of the 11 outer RBM1-RBM2 domains are modelled (labelled with black numbers) according to the density map but

are not built in the final structure.

b, Representative density maps of the outer RBM1 and RBM2 domains in the CCW-C ring.

c, Cross-section view of the MS ring with the embraced proximal rod and the export apparatus in the basal body-hook complex in the CCW state. The L1, L2 and L3 loops of FliF, which are extended from the MS ring, are highlighted in green, red and yellow, respectively.

d, Interactions of the 11 FliF peptide loops from the MS ring with the rod. The subunits of the proximal rod are colored and labeled as indicated. All residues (P309-N324) of L2 were precisely modeled in the C ring-containing basal body-hook complexes. The 11 loops are packed in the same right-handed helical manner as the rod subunits.

e, Representative density maps of the L1, L2 and L3 loops. The L3 loop is observed in both the basal body-hook complexes in the CCW and CW states. The model and density map of the L3 loop from the CW-biased motor are illustrated.

f, Detailed interactions of the L3 loop with the rod subunits. The interacting residues are shown as sticks.

g, Schematic diagram for the proposed mechanosensing mechanism of the bacterial flagellar motor and the potential key role of the C ring in the mechanosensing. The environmental mechanistic stimuli likely lead to the conformational changes of the filaments, hook and rod. It is possible that the 11 FliF peptide loops that are bound on the rod surface induce the conformational changes of the MS ring. Then the C ring adjusts the conformations of the $FliG_{CC}$ domains via the $Helix_{NM}$-$Helix_{MC}$-$FliG_{CN}$-$FliG_{CC}$ module cascade upon the conformational changes of the MS ring to regulate the loading of the stator units, as well as the recruitment of the CheY-P towards the C ring.
