# Supplementary material for: Structural basis of the bacterial flagellar motor rotational switching
Source: Cell Res. 2024 Aug 23;34(11):788–801. doi: 10.1038/s41422-024-01017-z (PMC11528121; doi:10.1038/s41422-024-01017-z)
Supplement: Supplementary file 6 — Supplementary information, Figure S6 [file 41422_2024_1017_MOESM6_ESM.pdf]

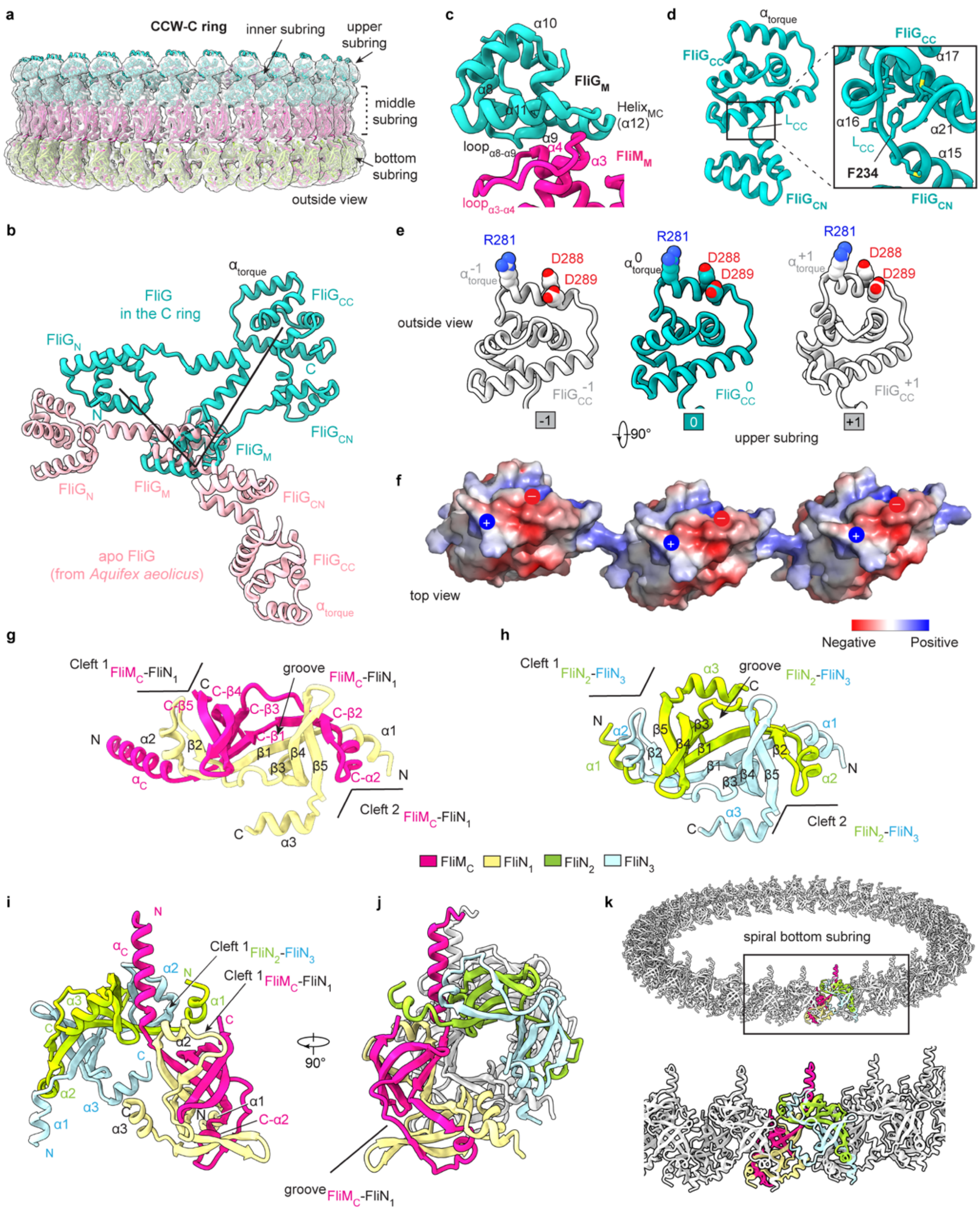

Supplementary information, Figure S6. The structure and inter-subunit interactions of the CCW-C ring.

a, The locally refined cryo-EM density map of the CCW-C ring with C34 symmetry. The four subrings of the CCW-C ring are labeled as indicated. The structural model of the CCW-C ring is colored as in Fig. 2a.

b, Structural superimposition of the structure of FliG in the CCW-C ring with the apo structure

of FliG from *A. aeolicus* (PDB ID: 3HJL) through the $FliG_M$ domains.

c, Detailed interactions of $FliG_M$ with $FliM_M$ in the protomer.

d, The detailed interactions of F234 with the $FliG_{CC}$ domain in the CCW-C ring.

e-f, Side view (**e**) of the conformation of the α_{torque} helices and top view (**f**) of the surface electrostatic potential of the upper subring in the CCW-C ring. The positively charged residue R281 and negatively charged residues D288 and D289 are shown as spheres and labeled as indicated (**e**). The surface colored in blue indicates the positively charged residues and red indicates the negatively charged residues in (**f**).

g-h, Structures of the $FliM_C$-$FliN_1$ heterodimer (**g**) and the $FliN_2$-$FliN_3$ homodimer (**h**). The $FliM_C$, $FliN_1$, $FliN_2$ and $FliN_3$ subunits are colored in red, wheat, green and light blue, respectively.

i, Inside view of the structure of the $FliM_C$-$FliN_{1\text{-}3}$ tetramer in the CCW-C ring.

j-k, Cross-section (**j**) and side (**k**) views of the spiral structure for the bottom subring of the CCW-C ring.
