# Supplementary material for: Structural basis of the bacterial flagellar motor rotational switching
Source: Cell Res. 2024 Aug 23;34(11):788–801. doi: 10.1038/s41422-024-01017-z (PMC11528121; doi:10.1038/s41422-024-01017-z)
Supplement: Supplementary file 7 — Supplementary information, Figure S7 [file 41422_2024_1017_MOESM7_ESM.pdf]

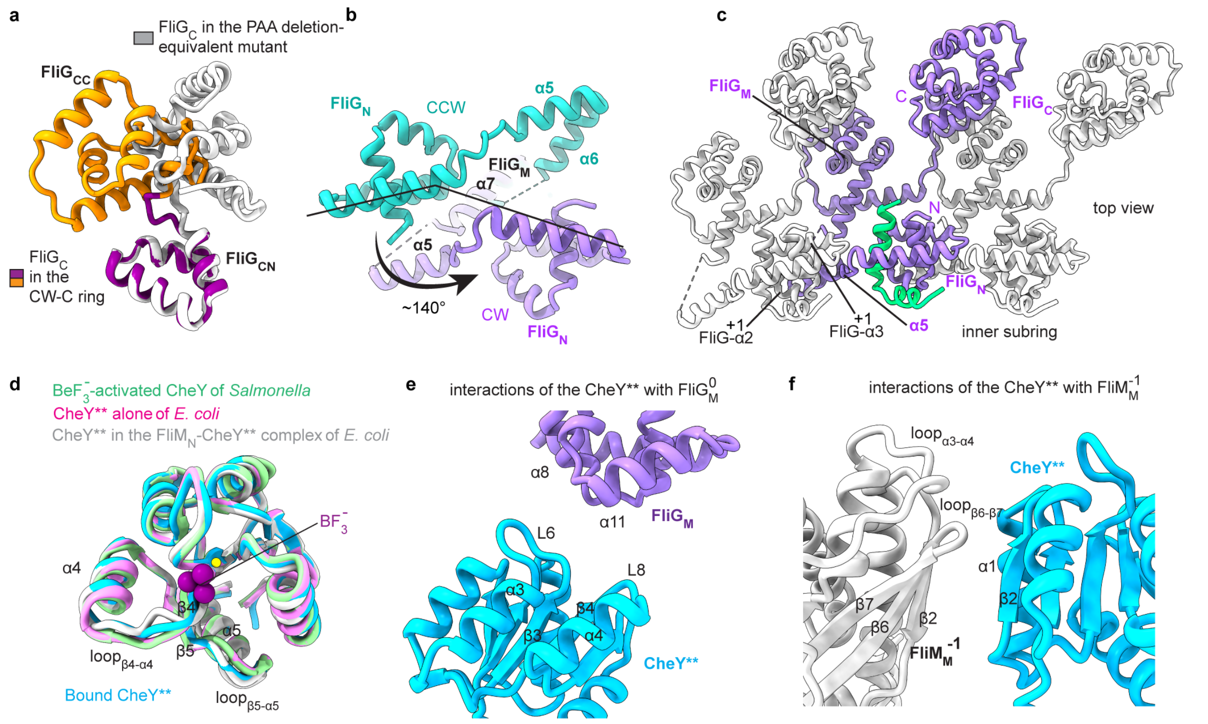

Supplementary information, Figure S7. The inter-subunit interactions of the CW-C ring.

a, Structural comparison of the $FliG_C$ domains in the CW-C ring and in the crystal structure of the $^{169}PAA^{171}$ deletion-equivalent mutant of FliG (PDB ID: 3AJC). The $FliG_{CN}$ and $FliG_{CC}$ domains in the FliG structure from the CW-C ring are colored in purple and yellow, respectively. The $FliG_C$ domain in the crystal structure is colored in grey.

b, Structural comparison of the FliG subunits in the CCW- and CW-C rings through structural superimposition via the $FliG_M$ domain. The conformational change of the $FliG_N$ domain is indicated by a black arrow.

c, Inter-subunit interactions of the FliG subunits in the CW-C ring. The $FliG_N$ domain extends its α5 helix to interact with the α2 and α3 helices of the $FliG^{+1}$ subunit of the next protomer.

d, Structural comparison between the CheY** in complex with C ring, the BF_3^--activated CheY from *S*. Typhimurium (PDB ID: 2FLW, the root mean square deviation (RMSD)=0.5 Å), the CheY** alone and in complex with $FliM_N$ (PDB ID: 1U8T, RMSD=0.73 and 0.63 Å, respectively) from *E. coli*. The structural models of CheY**, the BF_3^--activated CheY, the CheY** alone and the CheY** in the $FliM_N$-CheY** complex are colored in blue, green, pink and grey, respectively. The ligand BF_3^- is represented as purple spheres.

e, Detailed interactions of CheY** with $FliG_M$.

f, Detailed interactions of CheY** with the $FliM^{-1}$ subunit.
