# Supplementary material for: Structural basis of the bacterial flagellar motor rotational switching
Source: Cell Res. 2024 Aug 23;34(11):788–801. doi: 10.1038/s41422-024-01017-z (PMC11528121; doi:10.1038/s41422-024-01017-z)
Supplement: Supplementary file 8 — Supplementary information, Figure S8 [file 41422_2024_1017_MOESM8_ESM.pdf]

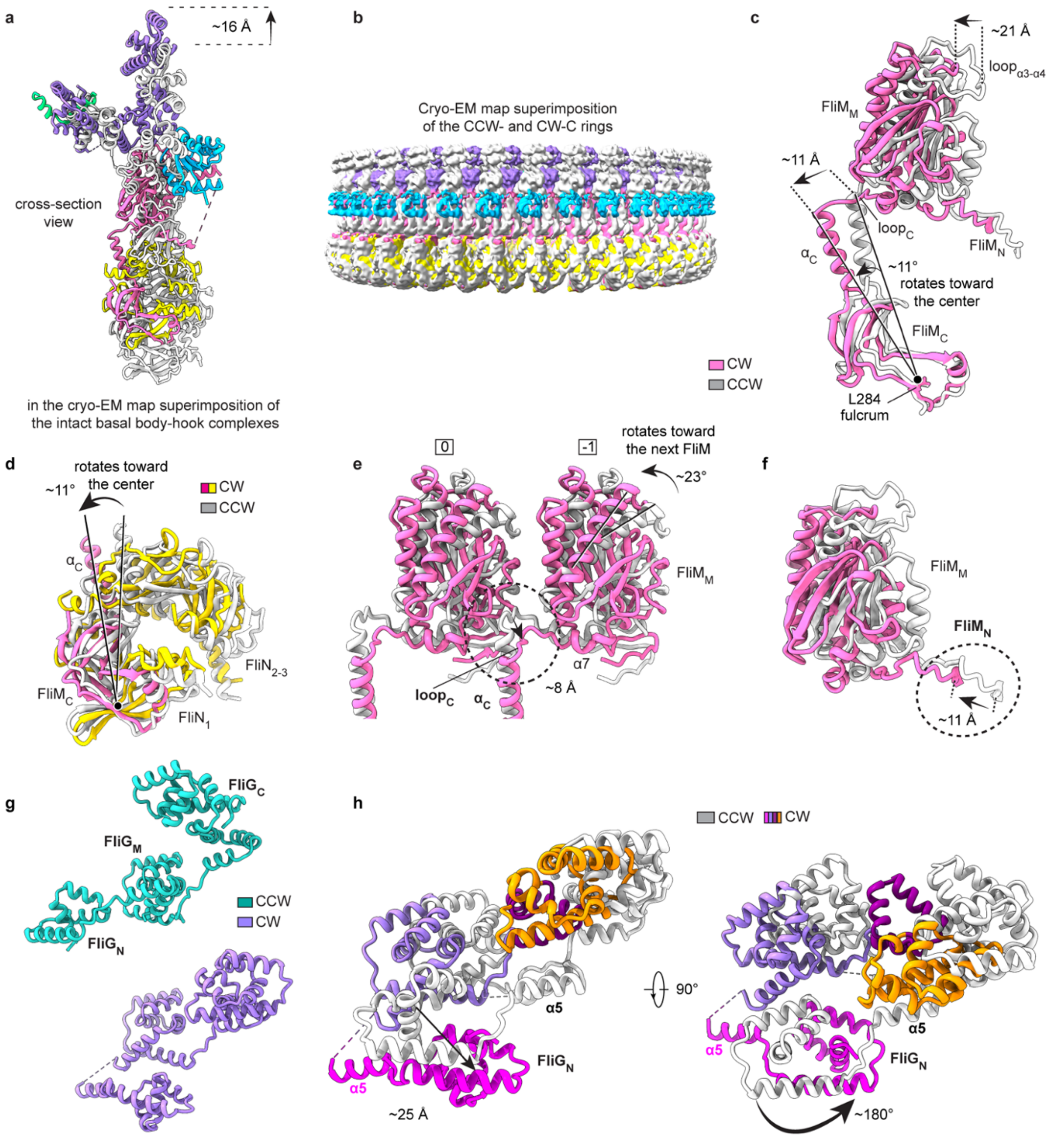

Supplementary information, Figure S8. The CheY-induced conformational changes of the C ring.**

a, Cross-section view of the structural comparison of the protomers of the CCW- and CW-C rings in the cryo-EM map superimposition of the basal body-hook complexes. The protomers in the CCW- and CW-C rings are colored in grey and as in Fig. 4a, respectively.

b, Side view of superimposition of the locally refined density maps of the CCW- and CW-C rings. The density map of the CCW-C ring is colored in grey. The density map of the CW-C ring is colored according to the CW-C ring model shown in Fig. 4.

c, The conformational changes of FliM in the structural superimposition of the CCW- and CW-

C rings.

d-e, The conformational changes of FliN subunits (**d**) and the $loop_C$ of $FliM_C$ (**e**) in the structural superimposition of the CCW- and CW-C rings. The FliN subunits from the CW-C ring are colored in yellow (**d**).

f, The conformational changes of $FliM_N$ domains. Conformational changes are indicated by black arrows and a dashed circle (**c-f**). The FliM subunits from the CCW- and CW-C rings are colored in grey and pink, respectively (**c-f**).

g, Structural comparison of the overall structures of FliG in the CCW- and CW-C rings. FliG in the CW-C ring (medium purple) has a more compacted conformation than that in the CCW-C ring (cyan).

h, Inside (left) and top (right) views of the domain rearrangement of FliG in the CCW- and CW-C rings. The $FliG_N$ domain rotates ~180° towards the prior protomer and moves downward by ~25 Å.
