# Supplementary material for: Structural basis of the bacterial flagellar motor rotational switching
Source: Cell Res. 2024 Aug 23;34(11):788–801. doi: 10.1038/s41422-024-01017-z (PMC11528121; doi:10.1038/s41422-024-01017-z)
Supplement: Supplementary file 9 — Supplementary information, Figure S9 [file 41422_2024_1017_MOESM9_ESM.pdf]

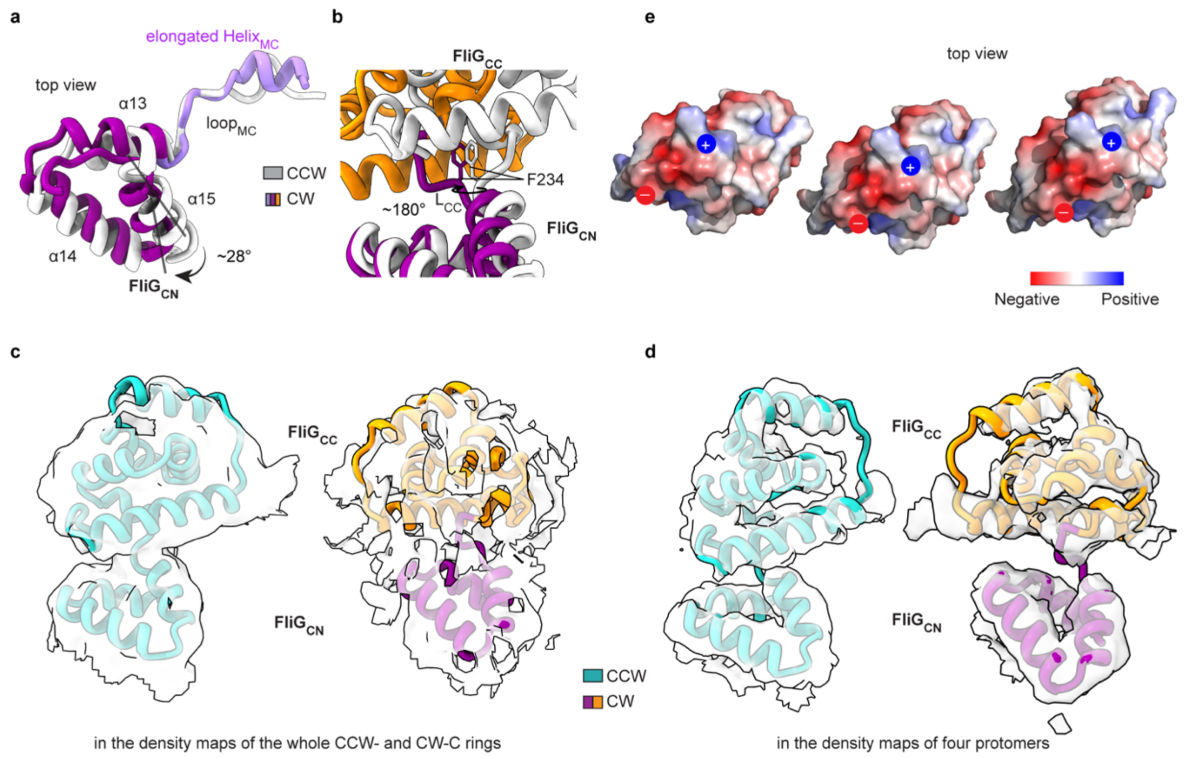

Supplementary information, Figure S9. The conformational changes and cryo-EM density maps of the FliG$_C$ domain.

a-b, Enlarged views of the CheY**-induced rotation of FliG$_{CN}$ on the surface of FliG$_M^{-1}$ (**a**) and the conformational changes of the hinge loop L$_{CC}$ (**b**) in the structural comparison in Fig. 5f.

c-d, Densities of the FliG$_{CN}$ and FliG$_{CC}$ domains of the CCW- and CW-C rings in the density maps of the C rings (**c**) and in the locally refined maps of four protomers (**d**).

e, The surface electrostatic potential of the upper subring in the CW-C ring. The surface colored in blue indicates the positively charged residues and red indicates the negatively charged residues.
