# Supplementary material for: Structural basis of the bacterial flagellar motor rotational switching
Source: Cell Res. 2024 Aug 23;34(11):788–801. doi: 10.1038/s41422-024-01017-z (PMC11528121; doi:10.1038/s41422-024-01017-z)
Supplement: Supplementary file 10 — Supplementary information, Figure S10 [file 41422_2024_1017_MOESM10_ESM.pdf]

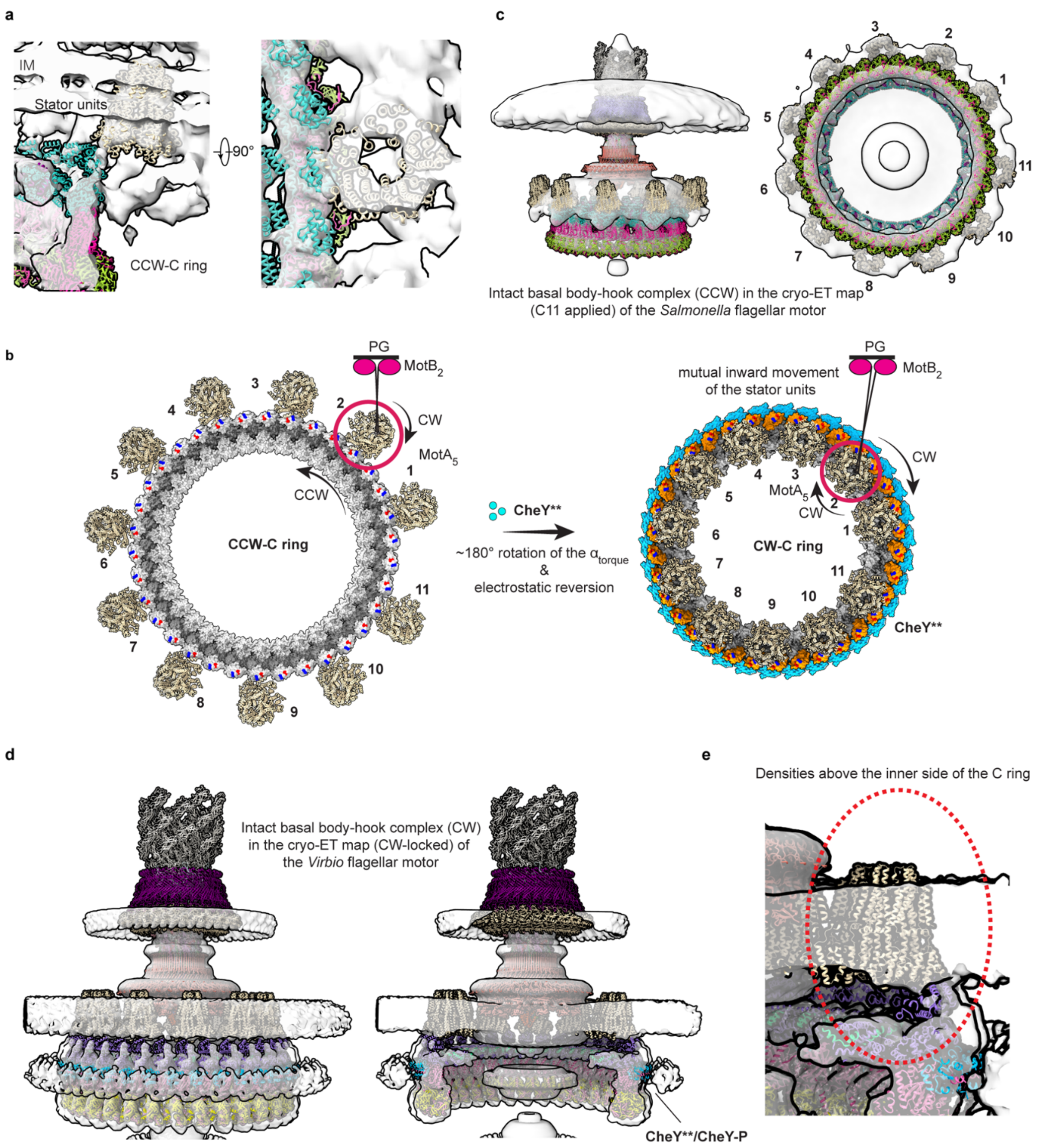

Supplementary information, Figure S10. The structural analyses of the relocation process of the stator units for rotational switching.

a, Cross-section (left) and top (right) views of structural modeling of the CCW-C ring and the $MotA_5$ pentamer of a stator of *S*. Typhimurium into the cryo-ET density map of the flagellar motor of *Helicobacter pylori* (EMDB: EMD-25123) in the default CCW state. The structural models of the CCW-C ring and the $MotA_5$ pentamer are colored as in Fig. 2a and wheat, respectively.

b, Structural models of the interactions of the C ring with 11stator units in the intact *Salmonella*

motors in the CCW (left) and CW (right) states.

c, The pseudo-atomic model of the intact basal body-hook complex containing CCW-C ring and 11 stator units in the cryo-ET density map (EMDB: EMD-3154) of the flagellar motor of *S*. Typhimurium. The side (left) and top (right) views are illustrated.

d-e, The protruding densities in the inner membrane, which are above the inner side of the upper subring of the CW-C ring, for accommodation of the stator units in the cryo-ET density map (EMDB: EMD-21837) of the flagellar motor of *V. alginolyticus* in the CW state. The pseudo-atomic model of the intact basal body-hook complex containing CW-C ring and 11 stator units, which was obtained in Fig. 6d, was fitted into the cryo-ET density map, and colored as indicated. The side (left) and cross-section (right) views are illustrated (**d**). The protruding densities that were ignored and not annotated in the previous study are highlighted in (**e**).
