# Supplementary material for: Structural basis of the bacterial flagellar motor rotational switching
Source: Cell Res. 2024 Aug 23;34(11):788–801. doi: 10.1038/s41422-024-01017-z (PMC11528121; doi:10.1038/s41422-024-01017-z)
Supplement: Supplementary file 11 — Supplementary information, Table S1 [file 41422_2024_1017_MOESM11_ESM.pdf]

Table S1. Cryo-EM data collection, model refinement and validation statistics of the intact basal body-hook complex in the CCW state.

	Protomers of the CCW-C ring	CCW-ring	LP ring	Proximal rod-export apparatus - 11 FliF loops
Components	$FliG_3$ $FliM_3FliN_9FliF_3$	$FliG_{34}$ $FliM_{34}FliN_{102}$ $FliF_{34}$	$FlgH_{26}FlgI_{26}$	$FliP_5R_1Q_4FliE_6FlgB$ $_5FlgC_6FlgF_5$ $L1_5\&L2_5\&L3_1$
EMDB	EMD-38546	EMD-39349	EMD-37618	EMD-37619
PDB	8XP0	8YJT	8WLE	8WLH
**Data collection & processing**				
Microscope	Titan Krios	Titan Krios	Titan Krios	Titan Krios
Magnification	81,000	81,000	105,000	105,000
Voltage (kV)	300	300	300	300
Electron exposure (e^-/ $Å^2$)	41	41	45	45
Defocus range (μm)	1.5 to 1.8	1.5 to 1.8	1.0 to 2.5	1.0 to 2.5
Pixel size (Å)	1.1	1.1	1.332	1.332
Symmetry imposed	C1	C34	C26	C1
Initial particles (no.)	19,627	19,627	22,192	22,192
Final particles (no.)	19,627	19,627	11,858	11,858
Map resolution (Å) (FSC = 0.143)	4.0	5.9	3.0	3.7
**Refinement**				
Initial model used (PDB)	3HJL, 4FHR, 5TDY, 4YXB, 4YX1, AF2	8XP0	7CBL	*ModelAngelo*
Model resolution (Å) (FSC = 0.5)	9.0	8.7	3.2	4.3
Map sharpening B factor ($Å^2$)	-150.8	-278.1	-112.3	-74.1
Model composition				
Non-hydrogen atoms	21,156	239,768	99,008	36,230
Protein residues	2,676	30,328	13,364	4,845
B factors ($Å^2$)				
Protein	81.81	81.81	64.04	53.53
Ligand	N/A	N/A	N/A	N/A
R.m.s deviations				
Bond length (Å)	0.002	0.003	0.004	0.005
Bond angles (°)	0.527	0.527	0.624	0.845
**Validation**				
MolProbity score	2.19	2.22	2.32	2.19
Clashscore	10.97	11.70	17.56	17.25
Ramachandran plot				
Favored (%)	95.56	95.56	97.32	96.13
Outliers (%)	0.80	0.80	0.20	0.46

	Distal rod with the hook	The whole rod with export apparatus and the hook	β-collar-RBM3 subrings of the MS ring (C34)
Components	$FlgG_{24}FlgE_{29}$	Rod, $FliF_{11}$ $FliP_5R_1Q_4FlgE_{29}$	$FliF_{34}$
EMDB	EMD-37627	EMD-37628	EMD-37620
PDB	8WLP	8WLQ	8WLI
**Data collection & processing**			
Microscope	Titan Krios	Titan Krios	Titan Krios
Magnification	105,000	105,000	105,000
Voltage (kV)	300	300	300
Electron exposure (e^-/ $Å^2$)	45	45	45
Defocus range (μm)	1.0 to 2.5	1.0 to 2.5	1.0 to 2.5
Pixel size (Å)	1.332	1.332	1.332
Symmetry imposed	C1	C1	C34
Initial particles (no.)	22,192	22,192	22,192
Final particles (no.)	11,858	11,858	11,858
Map resolution (Å) (FSC = 0.143)	3.8	3.8	3.2
**Refinement**			
Initial model used (PDB)	*ModelAngelo*	*ModelAngelo*	*ModelAngelo*
Model resolution (Å) (FSC = 0.5)	4.1	4.2	3.4
Map sharpening B factor ($Å^2$)	-71.4	-66.0	-116.2
Model composition			
Non-hydrogen atoms	131,528	167,758	43,350
Protein residues	17,770	22,615	5,576
B factors ($Å^2$)			
Protein	75.07	70.41	65.75
Ligand	N/A	N/A	N/A
R.m.s deviations			
Bond length (Å)	0.005	0.005	0.004
Bond angles (°)	0.780	0.795	0.707
**Validation**			
MolProbity score	1.95	2.08	1.57
Clashscore	13.94	17.25	11.33
Ramachandran plot			
Favored (%)	96.20	96.19	98.75
Outliers (%)	0.39	0.41	0.00

	MS ring with the proximal rod and the export apparatus (C1)	The membrane-anchored part (CCW)	The C ring-containing basal body-hook complex (CCW)
Components	$FliF_{34}FliE_6FliP_5FliR_1FliQ_4$ $FlgB_5FlgC_6$	Rod, Export apparatus, LP ring, MS ring, Hook	Intact basal body-hook complex
EMDB	EMD-37625	EMD-37630	EMD-37679
PDB	8WLN	8WLT	8WO5
**Data collection & processing**			
Microscope	Titan Krios	Titan Krios	Titan Krios
Magnification	105,000	105,000	81,000
Voltage (kV)	300	300	300
Electron exposure (e^-/ Å^2)	45	45	41
Defocus range (μm)	1.0 to 2.5	1.0 to 2.5	1.5 to 1.8
Pixel size (Å)	1.332	1.332	1.1
Symmetry imposed	C1	C1	C34
Initial particles (no.)	22,192	22,192	19,627
Final particles (no.)	11,858	11,858	10,318
Map resolution (Å) (FSC = 0.143)	4.3	4.1	7.4
**Refinement**			
Initial model used (PDB)	*ModelAngelo*<br>7CGO<br>AF2	7CBL, 7CGO<br>AF2<br>*ModelAngelo*	
Model resolution (Å) (FSC = 0.5)	7.3	6.6	
Map sharpening B factors (Å^2)	-74.6	-52.1	
Model composition			
Non-hydrogen atoms	99,060	338,664	
Protein residues	12,996	45,376	
Ligands	N/A	N/A	
B factors (Å^2)			
Protein	67.08	69.12	
Ligand	N/A	N/A	
R.m.s deviations			
Bond length (Å)	0.008	0.006	
Bond angles (°)	1.076	0.843	
**Validation**			
MolProbity score	2.37	2.28	
Clashscore	19.59	18.24	
Ramachandran plot			
Favored (%)	96.11	96.50	
Outliers (%)	0.90	0.48	
