# Supplementary material for: Structural basis of the bacterial flagellar motor rotational switching
Source: Cell Res. 2024 Aug 23;34(11):788–801. doi: 10.1038/s41422-024-01017-z (PMC11528121; doi:10.1038/s41422-024-01017-z)
Supplement: Supplementary file 12 — Supplementary information, Table S2 [file 41422_2024_1017_MOESM12_ESM.pdf]

Table S2. Cryo-EM data collection, model refinement and validation statistics for the intact basal body-hook complex in the CW state.

	**Protomers of the CW-C ring**	**The CheY**-bound CW-C ring**	**LP ring**	**The whole rod with export apparatus and the hook**
Components	$FliG_3FliM_3$ $FliN_9FliF_3$ $CheY^{**}_3$	$FliG_{34}FliM_{34}$ $FliN_{102}FliF_{34}$ $CheY^{**}_{34}$	$FlgH_{26}$ $FlgI_{26}$	Rod, $FliF_{11}$ $FliP_5R_1Q_4FlgE_{29}$
EMDB	EMD-38547	EMD-37570	EMD-37547	EMD-37601
PDB	8XP1	8WIW	8WHT	8WKK
**Data collection & processing**				
Microscope	Titan Krios	Titan Krios	Titan Krios	Titan Krios
Magnification	105,000	105,000	105,000	105,000
Voltage (kV)	300	300	300	300
Electron exposure (e^-/ Å^2)	50	50	50	50
Defocus range (μm)	1.2 to 1.8	1.2 to 1.8	1.2 to 1.8	1.2 to 1.8
Pixel size (Å)	1.2	1.2	1.2	1.2
Symmetry imposed	C1	C34	C26	C1
Initial particles (no.)	46,058	46,058	46,058	46,058
Final particles (no.)	28,119	28,119	24,190	24,190
Map resolution (Å) (FSC = 0.143)	4.4	5.6	2.8	3.3
**Refinement**				
Initial model used (PDB)	8XP0, 2FLW, AF2	8XP1	7CBL	*ModelAngelo*
Model resolution (Å) (FSC = 0.5)	7.9	10.2	2.8	3.6
Map sharpening B factor (Å^2)	-173.9	-264.3	-47.6	-46.2
Model composition				
Non-hydrogen atoms	24,297	275,366	99,008	167,771
Protein residues	3,084	34,952	13,364	22,617
B factors (Å^2)				
Protein	85.21	85.21	33.84	70.41
Ligand	N/A	N/A	N/A	N/A
R.m.s deviations				
Bond length (Å)	0.003	0.003	0.003	0.005
Bond angles (°)	0.579	0.579	0.556	0.765
**Validation**				
MolProbity score	2.15	2.17	1.58	1.80
Clashscore	8.52	8.96	11.62	11.96
Ramachandran plot				
Favored (%)	95.84	95.84	98.42	96.66
Outliers (%)	1.19	1.19	0.20	0.31

	β-collar-RBM3 subrings of the MS ring (C34)	MS ring with FlgB-Dc loops and FliE-α1 helices (C1)	The distal rod with hook
Components	$FliF_{34}$	$FliF_{34}$FlgB-Dc $loop_5$ FliE-α1 $helix_6$	$FlgG_{24}FlgE_{29}$
EMDB	EMD-37590	EMD-37595	EMD-37600
PDB	8WJR	8WK4	8WKI
**Data collection & processing**			
Microscope	Titan Krios	Titan Krios	Titan Krios
Magnification	105,000	105,000	105,000
Voltage (kV)	300	300	300
Electron exposure (e^-/ $Å^2$)	50	50	50
Defocus range (μm)	1.2 to 1.8	1.2 to 1.8	1.2 to 1.8
Pixel size (Å)	1.2	1.2	1.2
Symmetry imposed	C34	C1	C1
Initial particles (no.)	46,058	46,058	46,058
Final particles (no.)	24,190	24,190	24,190
Map resolution (Å) (FSC = 0.143)	2.9	3.7	3.3
**Refinement**			
Initial model used (PDB)	*ModelAngelo*	*ModelAngelo*	*ModelAngelo*
Model resolution (Å) (FSC = 0.5)	3.1	4.2	3.6
Map sharpening B factor ($Å^2$)	-95.5	-44.9	-48.2
Model composition			
Non-hydrogen atoms	43,350	44,690	131,528
Protein residues	5,576	5771	17,770
B factors ($Å^2$)			
Protein	65.75	65.74	75.07
Ligand	N/A	N/A	N/A
R.m.s deviations			
Bond length (Å)	0.004	0.004	0.004
Bond angles (°)	0.707	0.751	0.741
**Validation**			
MolProbity score	1.51	1.67	1.74
Clashscore	9.62	13.16	10.29
Ramachandran plot			
Favored (%)	98.75	98.70	96.68
Outliers (%)	0.00	0.00	0.30

	Proximal rod-export apparatus -11 FliF loops	The MS ring with export apparatus and proximal rod (C1)	The membrane-anchored part (CW)
Components	$FliP_5R_1Q_4FliE_6FlgB_5FlgC_6FlgF_5$ $L1_5$&$L2_5$&$L3_1$	$FliF_{34}FliP_5R_1Q_4FliE_6FlgB_5FlgC_6$	Rod, Export apparatus, LP ring, MS ring, Hook
EMDB	EMD-37594	EMD-37605	EMD-37611
PDB	8WK3	8WKQ	8WL2
**Data collection & processing**			
Microscope	Titan Krios	Titan Krios	Titan Krios
Magnification	105,000	105,000	105,000
Voltage (kV)	300	300	300
Electron exposure (e^-/ Å^2)	50	50	50
Defocus range (μm)	1.2 to 1.8	1.2 to 1.8	1.2 to 1.8
Pixel size (Å)	1.2	1.2	1.2
Symmetry imposed	C1	C1	C1
Initial particles (no.)	46,058	46,058	46,058
Final particles (no.)	24,190	24,190	24,191
Map resolution (Å) (FSC = 0.143)	3.3	3.8	3.4
**Refinement**			
Initial model used (PDB)	*ModelAngelo*	*ModelAngelo* AF2 7CGO	7CBL, 7CGO AF2 *ModelAngelo*
Model resolution (Å) (FSC = 0.5)	3.5	4.4	6.1
Map sharpening B factor (Å^2)	-48.0	-38.2	-28.7
Model composition			
Non-hydrogen atoms	36,243	99,073	338,677
Protein residues	4,847	12,998	45,378
B factors (Å^2)			
Protein	53.53	67.08	60.29
Ligand	N/A	N/A	N/A
R.m.s deviations			
Bond length (Å)	0.006	0.009	0.006
Bond angles (°)	0.845	1.086	0.815
**Validation**			
MolProbity score	1.80	2.29	1.92
Clashscore	10.74	17.75	13.81
Ramachandran plot			
Favored (%)	96.57	96.28	97.05
Outliers (%)	0.34	0.86	0.43

	The CheY** -bound basal body-hook complex (CW)
Components	Intact basal body-hook complex, $CheY^{**}_{34}$
EMDB	EMD-37684
PDB	8WOE
**Data collection & processing**	
Microscope	Titan Krios
Magnification	105,000
Voltage (kV)	300
Electron exposure (e^-/ $Å^2$)	50
Defocus range (μm)	1.2 to 1.8
Pixel size (Å)	1.2
Symmetry imposed	C34
Initial particles (no.)	46,058
Final particles (no.)	26,921
Map resolution (Å) (FSC = 0.143)	4.3
**Refinement**	
Initial model used (PDB)	
Model resolution (Å) (FSC = 0.5)	
Map sharpening B factor ($Å^2$)	
Model composition	
Non-hydrogen atoms	
Protein residues	
B factors ($Å^2$)	
Protein	
Ligand	
R.m.s deviations	
Bond length (Å)	
Bond angles (°)	
**Validation**	
MolProbity score	
Clashscore	
Ramachandran plot	
Favored (%)	
Outliers (%)	
